# Confidentiality, public interest, and the human right to science: when can confidential information be used for the benefit of the wider community?

**DOI:** 10.1093/jlb/lsad013

**Published:** 2023-06-14

**Authors:** Edward S Dove

**Affiliations:** School of Law, University of Edinburgh, Edinburgh EH8 9YL, UK

**Keywords:** confidentiality, confidential information, human right to science, public health, public interest, research

## Abstract

This article explores whether the human right to science can support the public interest as a legal basis to use and disclose confidential information. The contextual focus is scientific research; the jurisdictional focus is England. The human right to science, as reflected in the Universal Declaration of Human Rights (Article 27) and the International Covenant on Economic, Social and Cultural Rights (Article 15), hitherto has not been invoked in support of a public interest basis for lawful disclosure, but the argument is made herein that there may be scope to develop this jurisprudentially. On grounds of both law and policy, and in line with the underlying rationale of recent UK Government deployment of ‘COPI Notices’ for lawful use of confidential patient information in the course of the COVID-19 pandemic, I contend that the human right to science may well serve as a valuable juridical buttress to an overriding public interest justification to lawfully share confidential information. However, this could occur only in restricted circumstances where the public interest is clearly manifest, namely studies researching serious, imminent health threats to the general population that rely on confidential information accessed outside of existing statutory gateways, and not more routine scientific endeavors.

## I. INTRODUCTION

Individual-level information holds tremendous value for research organizations, healthcare systems, and commercial companies alike. Information gathered from individuals—be they patients, research participants, or otherwise—and be it in the form of health data, education data, socioeconomic data, political opinion data, or otherwise—helps contribute to better understandings of disease, public health threats, and human wellbeing more generally. These better understandings may, in turn, lead to lucrative, life-enhancing innovations in the form of diagnostics, therapeutics, vaccines, and devices—all of which hold even more tremendous value in the midst of a public health emergency. Additionally, and generally speaking, the more information that is obtainable in great volume, and the more information that is obtainable in identifiable form, the greater the value it holds, as there is better opportunity to make linkages across different data sets, to combine it with other data sets, run analyses, and to identify correlations and causations.^1^ There is considerable scientific and commercial imperative to gather information on a cumulative basis, both for its current and potential future value.

Cutting across this desire is a counterweight in the law, reflected in distinct but at-times overlapping legal regimes, namely confidentiality law, privacy law, and data protection law, all of which to seek to protect rights of individuals and limit uses of information relating to them. In this article, and in the interest of space, I focus only on confidentiality law and within the jurisdictional confines of England. Confidentiality law is a common law doctrine with strong roots in equity; privacy law, on the other hand, is an emerging regime in England, grounded both in human rights (in particular Article 8 of the European Convention on Human Rights, or ECHR) and in domestic jurisprudence, with the latter recognizing privacy (as one example) as meriting a stand-alone tort in the informational context only in 2004,^2^ and, north of the border in Scotland, as a domestic right only in 2019.^3^ Statute-driven data protection law has been in place in many countries around the world since the late 20th century, coincidental with the rise of automated systems (particularly computers) that process personal data; it seeks to protect against actions that can interfere with the fundamental rights of persons, including their generalized rights to privacy and to data protection.^4^ By contrast, confidentiality law holds that confidants (ie, recipients) of information, which has the necessary quality of confidence about it, are duty-bound to hold that information in confidence (that is, in secret), unless there is a lawful basis for disclosure, such as the consent of the confider. The doctor-patient relationship is a classic scenario in which the duty of confidence arises.^5^ It dictates that a duty is owed by the doctor to the patient in relation to almost all of the information disclosed in the course of encounters between these two parties (save for mere trivial tittle-tattle^6^).

The duty of confidentiality holds firm even as the traditional dyadic doctor-patient relationship has transformed in the 21st century, where the patient encounters a complex healthcare *system*, and research participants encounter a complex health research *system*, in which ‘the specialisation of care has resulted in the treatment details of each person needing to be shared between a team of practitioners’^7^ (and equivalent through research staff), and even as growing amounts of data are shared through multiple dispersed data sharing platforms, networks, and technologies. In each of these encounters with doctors, scientists, and the larger ‘system’, a duty of confidentiality potentially arises, although the legal landscape becomes increasingly unclear both with respect to who owes such a duty, the nature and extent of the duty, and the respective rights of the persons to whom the confidential information relates. As the amount of information obtained from individuals surges and is stored in an ever-increasing number of databases with linkages sought between them, questions arise regarding the extant legal and policy balance between the value of sharing confidential information for the benefit of the wider community and the value of protecting such information on both intrinsic and instrumental grounds.

In this article, I want to focus on a particular basis in confidentiality law that would—in principle—allow for the lawful use and disclosure of various types of confidential information for secondary use purposes,^8^ such as scientific research, that could be seen as a benefit for the wider community. This basis is known as the *public interest*. As this article will discuss, the public interest is a nebulous concept that shapeshifts over the years as underlying social values and norms evolve in society.^9^ Nonetheless, it retains coherence and meaning and has received jurisprudential treatment (as well as doctrinal analysis and insight from professional guidance) that helps aid our analysis of its workings. Much of that treatment has come through interpretations of the duty of confidentiality and the allied and emerging tort of misuse of private information as viewed through the lens of the ECHR, notably Article 8 (right to privacy), frequently read in tandem with Article 10 (freedom of expression)—not to mention their limitations under Articles 8(2) and 10(2), respectively—and both of which have been domesticated in the UK’s Human Rights Act 1998 (HRA 1998). Deeper understanding of the public interest is also beginning to emerge from academic commentary on the interpretation of the United Kingdom’s Data Protection Act 2018, which introduces a new public interest test applicable to the scientific research processing of personal health data.^10^

What has not received any consideration to date, however, is whether there are *other* normative instruments, including other human rights instruments ostensibly with some legal effect in the UK, such as the International Covenant on Economic, Social and Cultural Rights (ICESCR)—and even more hortatory instruments such as the Universal Declaration of Human Rights (UDHR)—that could have a bearing on the interpretation and scope of the public interest as a basis to justify the use and disclosure of confidential information. This is worthy of exploration for two reasons. First, it raises significant potential (at least at first glance) for expanding the circumstances in which confidential information can be used for the benefit of the wider community—even in the absence of consent signaled from the rights-bearing confider. Second, it brings much-needed clarity to the interplay between confidentiality, the public interest, and human rights—including those that are not incorporated in the HRA 1998.

In what follows, I examine the extent to which one specific article in two legal instruments, the ICESCR and UDHR, may be lawfully invoked to help ground a public interest basis for the lawful disclosure of confidential information in certain circumstances. This is Article 15(1)(b) and Article 27(1), respectively—each of which speaks to ‘the right of everyone to enjoy the benefits of scientific progress and its applications’ (ICESCR) and ‘to share in scientific advancement and its benefits’ (UDHR). In shorthand, and colloquial language, this is frequently referred to as the ‘human right to science’. Thus, the key question at the heart of this article becomes this: can a confidant (data custodian)—a person or institution^11^ who otherwise is obliged not to disclose confidential information they have received—lawfully share a person’s confidential information for a secondary purpose, namely a scientific research purpose, without that person’s consent, on a public interest basis? The foundation of an argument to this effect would be grounded in a claim that there is an overriding public interest that the information ought to be used for research into a matter that is of wide benefit to the community and in so doing can (help) enable everyone to enjoy the benefits of scientific progress and its applications. Put more simply: just how far, if at all, can we stretch the public interest basis in the law of confidentiality, and to what extent might the human right to science assist?

The question is not merely an academic exercise. On a daily basis, data custodians must ensure that they are adhering to ethical and legal obligations in their work with identifiable (and non-identifiable) information. In some jurisdictions, while consent might operate as the primary legal basis to use and disclose confidential information, especially for non-direct care purposes such as research, there are many circumstances in which this may be neither possible nor the most appropriate basis. Parliament itself has recognized this, creating some years ago a statutory gateway applicable in England and Wales, known as ‘section 251 approval’, to permit disclosure and use of ‘confidential patient information’ for a ‘medical purpose’ (including ‘medical research’), on a public interest basis, without consent^12^ (as will be discussed). Northern Ireland has also recognized this, passing legislation that enables the use of health and social care information which identifies individuals to be used for health care or social care purposes which are in the public interest, without the consent of the individuals whose information may be used.^13^ Circumstances in which consent may be neither possible nor the most appropriate basis include use of information that relates to persons who have since died (the obligation of confidentiality continues for some time after death^14^), and where it would be impracticable to obtain the consent from a massive cohort of individuals. Indeed, many in the public health research community prefer to make available and have access to individual-level information for secondary uses in ways that respect ethical and legal norms, but not necessarily through the prism and paradigm of consent.^15^ This is especially the case with large-scale data linkage projects and use of data that was originally collected many years prior. As Adams and colleagues note: ‘Seeking consent is often problematic in data-intensive research because the linkage and extraction of data are remote in time and place from the original collection of data in the healthcare facility.’^16^ Outside the scope of explicit consent for use of information within a specific research project, ‘[r]eliance on extended or unspecified consent, or on implied consent, leaves the person disclosing the information vulnerable to an action for breach of confidence because of the uncertainty about the validity and scope of the consent.’^17^

Many scholars have noted the drawbacks and weaknesses with consent operating as a (primary) legal basis in this area, including, *inter alia*, difficulty obtaining truly informed consent (eg, information overload and complexity for individuals providing their consent, difficulty tracing all individuals to obtain their consent); selection bias; and the failure of consent (or wrongful belief in consent) to act as an adequate—much less complete—safeguard for the privacy and safety of information stored in databases.^18^ Outside the existing statutory gateway for disclosing ‘confidential patient information’, were data custodians able to lawfully disclose and use other kinds of confidential information *without* having to seek and obtain the consent of the person to whom the information relates, and instead rely on the basis of an overriding public interest, it would enable a more cost-effective, efficient, as well as unprecedented level of access to information and thereby create a boon for science. But it would likely also generate concern for the adequate protection of confidential information of individuals availing themselves of various services, be they in health, employment, or education. Thus, beyond the primary legal question phrased above, a more socio-political question also arises: is the public interest legal basis, to enable confidential information sharing without consent for research purposes, one that is considered reasonable and acceptable by society, and if so, for which specific kinds of research purposes? This question concerns matters of political legitimacy, social acceptability, and social license; it is a question that I cannot hope to address in this article, which focuses on the legal question, but I acknowledge that social, political, and ethical values are critically important and would also have to be addressed to fully flesh out this issue.

To answer the legal question, the article is organized as follows. In Section 2, I provide a brief primer on confidentiality law and its centuries of jurisprudential development. In the medico-scientific context, confidentiality protects not only the information disclosed by a patient to their doctor and healthcare team, but also, in more recent years, by a research participant to the research staff. In both instances, the relationship may be governed by a contractual or equitable obligation owed to the confider. I demonstrate here that the paradigmatic framing of a doctor-patient relationship is not, in fact, a true reflection of the actual or potential nature and limits of the duty of confidence, as seen for instance in the context of a researcher-participant relationship (not to mention employer-employee relationships), and this therefore may start to open to the door to a possibly broader public interest argument.

In Section 3, taking up the interim conclusion in Section 2, I briefly cover the available legal bases to lawfully share confidential information, focusing on the public interest basis. Looking at jurisprudence, academic commentary, legislation, regulatory guidance, and (albeit limited) public opinion polling, I argue that the categories that fall within this basis are broader than we may think; there is some jurisprudential, doctrinal, and public openness to going beyond ‘traditional’ categories of the public interest, namely risk of serious harm or risk of serious crime.

Section 4 then analyzes the human right to science and the extent of its domestic implementation and jurisprudential take-up in the UK, exploring the potential for this human right to serve either as a right on its own that conflicts with a ‘right’ of confidentiality such that it may override the latter, or, as I go on to suggest, more of a juridical ‘buttress’ to a public interest basis for confidential information disclosures.

I then turn in Section 5 to discuss two hypothetical scenarios as a means to consider whether the public interest may be expanded to permit a lawful breach of confidentiality with the added reinforcement of the human right to science. I argue that this is indeed possible, but likely only in research scenarios involving serious, imminent health threats to the general population, such as public health emergencies, as this is a purpose that would most clearly meet a common understanding of the public interest and reflects a ‘net interest’ in which all members of the public have in common. Support for my argument that the public interest is an appropriate legal basis for use of confidential information limited to this context is garnered from the recent UK Government deployment of ‘COPI Notices’ during the COVID-19 pandemic. It is worth noting here that while England is this article’s jurisdictional focus, these two hypothetical scenarios are of wider significance for other common law jurisdictions (not to mention civil law jurisdictions which also recognize a duty of confidence).

Finally, in Section 6, I bring the analysis together to conclude that ultimately, while the scope of the public interest in confidentiality law is indeed flexible, and some forms of scientific research may fulfil the necessarily strict criteria to permit lawful disclosure in the absence of consent, the very nature of the public interest serving as an exception to the duty means that caution and precaution must be the guardians of any expansion. The ‘hook’ of the human right to science would buttress a public interest legal basis to override the duty of confidentiality, but in England, given limited recognition of human rights not explicitly domesticated in law, the human right to science could not serve in its own right as a justiciable right that might override and come into conflict with a common law right and civil right such as confidentiality (and, more arguably, privacy^19^). Yet even in serving as a buttress, recognizing the human right to science within confidentiality law could provide firmer, more robust justification for disclosures grounded in an overriding public interest. This said, I argue that for most kinds of scientific research outside the confines of a public health emergency, the public interest basis would be of limited to no utility and another lawful basis would be required to use and disclose confidential information. Invariably that will—or should—equate to obtaining either section 251 approval to the extent the context is ‘medical research’ that involves ‘confidential patient information’, or otherwise obtaining the explicit consent of the individuals involved. This demonstrates both the inherent limits of international human rights generally, especially as they are treated in domestic courts, as well as a rather cautious approach and interpretation to the common law, which interestingly appears more pronounced in England than in other jurisdictions, including those within the UK.^20^

## II. CONFIDENTIALITY LAW: A BRIEF PRIMER

The law of confidentiality is an ancient doctrine in England, incrementally developed by courts over the centuries and in recent times, modified to some degree by statute law (as discussed below). Unlike in the USA, the doctrine has long been viewed as a core part of private law obligations.^21^ Some of its elements related to good faith and conscience may be traced to the early courts of equity, but firmer roots began to appear in 18th-century jurisprudence.^22^ Its core elements are seen in cases such as *Abernethy v Hutchinson*^23^ and *Prince Albert v Strange*,^24^ where English courts recognized that people may have a legal duty—be it implied or explicit—under trust or confidence (together comprising obligations arising in equity), or contract, to not disclose or otherwise make available information (or material) that has the necessary quality of confidence about it, in the absence of a lawful basis.^25^ In more recent times, courts have explicitly recognized the public interest basis of the duty of confidence. In *Attorney-General v Guardian Newspapers (No 2)* (often called the *Spycatcher* case), Lord Griffiths noted that duty rests on the ‘the public interest in upholding the right to confidence, which is based on the moral principles of loyalty and fair dealing’, while Lord Goff stated that ‘the basis of the law’s protection of confidence is that there is a public interest that confidences should be preserved and protected by the law’.^26^

The modern formulation of the elements necessary to establish a cause of action for breach of confidence, as reflected in a now-modified tripartite test, can be traced to the judgment of Justice Robert Megarry in *Coco v AN Clark (Engineers) Ltd*,^27^ wherein he stipulated that:

First, the information itself… must ‘have the necessary quality of confidence about it’. Secondly, that information must have been imparted in circumstances importing an obligation of confidence. Thirdly, there must be an unauthorised use of that information to the detriment of the party communicating it.^28^

It may be taken as almost axiomatic that information disclosed in the context of a doctor-patient relationship will have the necessary quality of confidence about it, and is imparted in circumstances importing an obligation of confidence. Indeed, as far back as the 1851 Scottish case of *AB v CD*,^29^ it was stated:

… that a medical man, consulted in a matter of delicacy, of which the disclosure may be most injurious to the feelings, and possibly, the pecuniary interests of the party consulting, can gratuitously and unnecessarily make it the subject of public communication, without incurring any imputation beyond what is called a broach of honour, and without the liability to a claim of redress in a court of law, is a proposition to which, when thus broadly laid down, I think the Court will hardly give their countenance.^30^

It is well-known (and thus no more need be said about it) that doctors have a moral and legal duty not to disclose information about their patients to others unless there is a stronger countering duty. What is more important for the purposes of this article is the recognition that the duty arises in other situations within the medico-scientific context. As Richards and Solove note, the English law of confidentiality ‘is much more open-ended in the relationships it protects’;^31^ the court in *Stephens v Avery* affirmed that ‘the relationship between the parties is not the determining factor. It is the acceptance of the information on the basis that it will be kept secret that affects the conscience of the recipient of the information’.^32^

Regarding the first two elements of the test, then, though there is little written on the subject from a legal perspective, a duty of confidence also may be seen to arise between a research participant and a researcher, as well as with a data custodian who holds information for safeguarding and for research-related and other purposes. In these scenarios, information having the necessary quality of confidence may be obtained in the course of a research project, such as health information obtained from a survey, longitudinal study, or observational study. This confidentiality obligation is commonly evidenced explicitly, rather than impliedly, in participant information sheets,^33^ where researchers commit to treat the information provided by the participant to them as confidential, and seek to anonymize and otherwise not disclose the information without the prior consent of the participant, or unless there is another lawful basis for doing so. Likewise, data custodians commit to holding identifiable information in confidence through a variety of safeguards.^34^ While we should be encouraged to see the duty in its entirety, beyond the ‘traditional’ relationships such as doctor-patient, we should also be mindful that the nature of the duty and its exceptions may differ as between, for example, the therapeutic (clinical) context where use of the information may have more direct benefit for the individual and the non-therapeutic research context, where use of the information may have more indirect benefit for the individual and instead carries social value for the wider community. I return to this consideration later in the article.

The last element to establish a claim for breach of confidence, concerning *unauthorized* use of that information to the *detriment* of the party communicating it, is necessarily dependent on the scope of the duty owed in the particular circumstance. As Simon Brown LJ opined in the case of *Source Informatics*, the test is whether a reasonable person’s conscience would be troubled by the proposed use of the relevant information.^35^ Does this mean that the misuse, actual or threatened, must be detrimental to establish a breach of confidentiality? In the case of private confidences, such as that between a doctor and patient or between a research participant and the researcher, we may safely assume that a confider likely will retain an interest in the information being kept confidential and not further disclosed to others, *regardless* of whether disclosure would be positively harmful to them, for reasons which may be perfectly understandable. In the medico-scientific context, it has long been recognized that health information is among the most sensitive of personal information and any use and disclosure of that information that is not authorized by the confider is reasonably likely to be detrimental to the confider (be it a concern of harm to autonomy interests, dignity, moral distress, or pecuniary damage).^36^ In any event, this third element has been modified in more recent jurisprudence, pulling away from the need to establish detriment, such that it must be satisfied that the confidential information has been misused or is threatened to be misused.^37^ Misuse can arise when a person merely accesses and acquires confidential information, such as intentionally looking at a patient’s medical notes, even if there is no disclosure per se from the doctor to the snooping person.^38^

Thus, we may assume that in the medico-scientific context, including both therapeutic (eg, doctor-patient) and non-therapeutic (eg, researcher-participant) contexts, a duty of confidentiality is *prima facie* established and owed by the confidant to the patient-participant confider in relation to the information^39^ that is disclosed by them, and, in the absence of any authorization by them to disclose or otherwise use that information, a *prima facie* cause of action for breach of confidence will be established. Again, however, the nature of the duty and its exceptions may have nuances of difference between the therapeutic and non-therapeutic contexts, and we thus ought to be attuned to the potential for these differences to lead to different outcomes when it comes to assessing how the duty is operationalized—and the circumstances in which it may be lifted lawfully.

Indeed, as has already been hinted at above, it is well-established that the duty of confidentiality is not absolute. In addition to a defendant-confidant countering each of the three elements to establish the cause, several limits, defenses, or bases (I use these terms interchangeably) are available that justify the lawful disclosure of confidential information. These include: (i) disclosure that is required or authorized by statute; (ii) consent by the confider to the disclosure (with the consent being either explicit or implied, general or specific); (iii) the inherent jurisdiction of the court (eg, the power of a court to order discovery); (iv) privilege (eg, Parliamentary or judicial proceedings); (v) evidence that the confidant was already in prior possession of the information before disclosed to them by the confider, or subsequently acquired the information by independent (and lawful) means; and, most importantly for the purposes of this article, (vi) the existence of an overriding public interest basis for the disclosure such that the balance weighs in favor of disclosure.^40^

Regarding this last basis, we have seen already that confidentiality law itself is seen as being grounded in a public interest that confidences should be preserved and protected by the law. In the health context, English courts have recognized that ‘[t]here is a strong public interest in respecting medical confidentiality which extends beyond the privacy of the individual patient’,^41^ and have quoted favorably the European Court of Human Rights case *Z v Finland*, in which the Court held that:

Respecting confidentiality of health data is a vital principle in the legal systems of all the Contracting Parties to the Convention. It is crucial not only to respect the sense of privacy of a patient but also to preserve his or her confidence in the medical profession and in the health services in general. Without such protection, those in need of medical assistance may be deterred from revealing such information of a personal and intimate nature as may be necessary in order to receive appropriate treatment and, even, from seeking such assistance, thereby endangering their own health and, in the case of transmissible diseases, that of the community.^42^

Yet, in certain circumstances, confidentiality may be overridden where there are sufficient countervailing public interests favoring disclosure. What are these circumstances? I begin to chart this below. As will be seen, the circumstances are neither finite nor foreclosed; one doctrinal text on confidentiality law has commented, ‘[t]he scope in English law of what has come to be referred to as the “public interest defence” has broadened over time.’^43^ The question this article asks is: if the scope of the public interest has broadened over time, just *how broad* has the scope become such that a confidant may lawfully share confidential information—even in the absence of consent? I turn to this question in Section III as means to then draw out the argument that the human right to science can help support the legal basis for disclosure, albeit in limited circumstances.

## III. THE PUBLIC INTEREST AS A BASIS TO PERMIT A LAWFUL BREACH OF CONFIDENTIALITY

In Section 3, I briefly cover the available bases to lawfully share confidential information, focusing on the public interest basis and demonstrate how the categories that may fall within this basis are broader than we may think and that much of existing jurisprudence has indicated to date. While much ink has been spilled on the concept of the public interest^44^ and how it might be established as a meaningful construct,^45^ relatively little has focused on its application to confidentiality law. This is a normative gap that ought to be addressed. As Feintuck warns: ‘Though the very phrase “the public interest” has an air of democratic propriety, the absence of any identifiable normative content renders the concept insubstantial, and hopelessly vulnerable to annexation or colonization by those who exercise power in society.’^46^ I take up the view that the public interest may be seen as ‘any action which is conducive to the fulfilment of goals which the public wants for itself as a whole’^47^ and further find value in Taylor and Whitton’s expression that proper invocation of the public interest (in the data protection law context) requires ‘respect for persons as free and equal members of society’ and ‘requires that the adoption of any trade-off between common interests is justified in terms that are both accessible and acceptable to them’.^48^ This puts us on more solid conceptual footing and has a good deal of resonance in the confidentiality law context, too, and as such will be further explored in a later section charting the circumstances in which the public interest legal basis may have purchase. I begin with tracing the evolution of the public interest basis in confidentiality before turning to its particular application in the research context.

### III.A. The Evolution of the Public Interest Basis

The origins of the public interest basis lie in what has been called the defense of iniquity. As Sir William Page-Wood VC observed in the 1856 case of *Gartside v Outram*,^49^ there can be ‘no confidence as to the disclosure of iniquity’, in this case being the disclosure of falsified sales notes to deceive customers. In other words, when the information concerns a risk of serious public harm, the information would be regarded both at common law and in equity as lacking the necessary attribute of confidence to forbid such disclosure. The facts of a case would justify disclosure because the confider has behaved disgracefully or criminally such that it would be in the public interest that their behavior, or at least the underlying information, should be exposed. Well through the 1980s, jurisprudence reflected a narrow interpretation of public interest disclosure along the lines of crime, national security, and iniquity. As a 1984 Scottish Law Commission report on confidentiality law summarized:

It would be fair to say, by way of summary, that the English courts have been reluctant to concede that there is a public interest in breaching confidence—except where the information relates to crime or national security or to some form of misconduct—and, with very rare exceptions, have been reluctant to permit disclosure otherwise than to a public official such as a police officer. A right to disclose on the part of the press is scarcely recognised.^50^

In the past few decades, however, the defense (or basis) has been broadened to account not only for cases of serious misdeeds, but also cases where, *inter alia*, there is danger of serious harm.^51^ Again, to quote from the Scottish Law Commission:

…the public interest defence should not, in our view, be confined to what may be regarded as ‘iniquity’ or ‘misconduct’, nor should there be pre-determined constraints on the range of persons to or by whom information may, in suitable circumstances, be disclosed, despite the existence of an obligation of confidence. […] This is not to say that disclosure is appropriate in every case. All we are saying here is that a defender must not be precluded from arguing that such disclosure whether to a public official or otherwise is justifiable in the particular circumstances of the case.^52^

More recent jurisprudence reflects a principle that confidentiality law permits the duty to be overridden on a case-by-case basis. It explicitly sets a high bar for this, however, because of the importance of considering not just the harms that may be mitigated in a particular case by disclosure of confidential information balanced against harm to the individual’s trust and engagement, but also because of the fundamental importance of protecting public confidence in infrastructures and wider systems, such as the healthcare system and health research system. Indeed, the bar has been so high that the public interest basis has been formulated as a ‘requirement’, as per Lord Goff’s encapsulation in the *Spycatcher* case:

… although the basis of the law’s protection of confidence is that there is a public interest that confidences should be preserved and protected by the law, nevertheless that public interest may be outweighed by some other countervailing public interest which favours disclosure. This limitation may apply […] to all types of confidential information. It is this limiting principle which may require a court to carry out a balancing operation, weighing the public interest in maintaining confidence against a countervailing public interest favouring disclosure.

Embraced within this limiting principle is, of course, the so called defence of iniquity. In origin, this principle was narrowly stated, on the basis that a man cannot be made ‘the confidant of a crime or a fraud’ … *But it is now clear that the principle extends to matters of which disclosure is required in the public interest* … .^53^

As commentators have noted, the limiting principle of only such form of disclosure as the public interest *requires* is:

… consistent with the underlying notion of confidentiality as an obligation of conscience in recognising that there may be circumstances in which a conscientious recipient of confidential information would reasonably consider it right as a responsible citizen to make some form of disclosure of the information.^54^

In such cases, it may be said that no obligation of confidence exists in contract, equity, or other jurisdictional basis insofar as the subject matter concerns a risk of serious public harm (including but not limited to cases of ‘iniquity’).

We might also consider there to be a useful distinction between a disclosure which may not ‘trouble the conscience’ of the confidant (as per *Source Informatics*,^55^ discussed below)—such as the disclosure only of anonymized information—and the disclosure of information in circumstances where a conscientious recipient would consider it right as a responsible citizen to disclose (ie, the distinction between disclosure being permitted and it being required as a matter of conscience). Further, we might query what would trouble the conscience of the conscientious data custodian (rather than, say, the conscientious doctor). Might reasonable considerations of benefits from science, and the right to science, also factor into the data custodian’s conscience? And if so, should that matter? What if they themselves are a scientist? Should a conscience test apply in determining whether there is a breach of confidence if, as we might surmise, most scientists will think that their research projects are (invariably) a good thing and thus sharing confidential information to further them may well not trouble their conscience? I continue to unpack this exploratory thread below.

Before doing so, however, it is necessary to chart the extant, general categories to which this public interest basis for use or disclosure has purchase. These include (i) risk of serious harm to others and serious crime; (ii) national security; (iii) the administration of justice; and (iv) a matter of comparable public importance such that it may fairly be regarded as necessary in the public interest that a person possessing such information should be free to disclose it to an appropriate third party, whether or not the matter involves individual wrongdoing (by the claimant or anyone else).^56^ In this article, I focus on the first category, although the fourth category also may be of some relevance to the analysis with respect to the broadness of a matter ‘of comparable public importance’.

### III.B. Risk of Serious Harm to Others and Risk of Serious Crime

As noted above, the bar has been set high for a countervailing public interest which favors disclosure, and in the medico-scientific context, this has been interpreted as preventing risk of serious harm to others (both physical and psychological) and preventing or detecting serious crime. Two principal sources evidence this claim: case law and professional regulatory guidance, the latter of which holds considerable persuasive force in medical jurisprudence. Both suggest that public interest, at least to date, has been interpreted as permitting disclosure of confidential information if the benefits to an individual or society outweigh both the public and the participant-patient’s private interest in keeping the information confidential—but in specific instances involving some sort of risk of serious harm.

The key case in this area is *W v Egdell*.^57^ The plaintiff, W, shot and killed five people and injured two others. He pleaded guilty to manslaughter on the ground of diminished responsibility and was ordered to be detained in a secure hospital on the grounds of him suffering from paranoid schizophrenia. At the time of the action for breach of confidence, W was compulsorily detained in a secure hospital but was being considered for transfer to a regional secure unit, as a step toward eventual release back into the community. When a recommendation for transfer was refused, W took steps to apply to a mental health review tribunal for conditional discharge. To that end, his solicitors instructed Dr Egdell, a consultant psychiatrist, to report on W’s mental state. His report conflicted substantially with that of W’s own medical advisers and he recommended further investigation of this conflict in opinion. Further, he recommended that attention should be given to other information, including W’s confession that he had a continuing and long-standing interest in explosives, which apparently had not been noted in other reports. W subsequently withdrew his application and his solicitors refused to forward Dr Egdell’s report to those responsible for his care and for any future recommendations as to his transfer to a less secure facility or discharge. Dr Egdell nonetheless sent a copy of his report to the assistant medical director at the hospital and also pressed for a copy to be sent to the Home Office to be considered by those responsible for reviewing W’s case. W brought an action against the defendant alleging breach of confidence.

The Court of Appeal held that in carrying out a balancing operation, weighing the public interest in maintaining confidence against a countervailing public interest favoring disclosure, each court must reach its own decision on the balance. However, in doing so, it is legitimate for the court to give ‘such weight to the considered judgment of a professional [person] as seems in all the circumstances to be appropriate’,^58^ in this particular case, Dr Egdell. On the facts, that balance ‘clearly lay in the *restricted disclosure* of vital information to the director of the hospital and to the Secretary of State who had the onerous duty of safeguarding public safety’.^59^ In both this case and an earlier case of *X v Y*,^60^ the courts paid great heed to the advice provided by the professional regulator (the General Medical Council, or GMC) contained in its ethical guidance to doctors,^61^ but the balance ultimately remains one for the courts and not for professional bodies or the government to decide.

In the more recent case of *ABC v St George’s Healthcare NHS Trust*,^62^ the High Court ruled that in considering whether the interests of public health or safety should permit a doctor to disclose information given in circumstances importing an obligation of confidentiality (in this case, information concerning the possibility of the patient’s daughter having Huntingdon’s disease), a doctor may owe a third person who is in a close proximal relationship with the patient a duty of care to balance their interest in being informed of their genetic risk against the patient’s interest *and the public interest* in maintaining confidentiality. The scope of that duty extends to conducting a balancing exercise between the interests of the patient and the at-risk third person, and to acting in accordance with its outcome. Some legal commentators have stated that ‘it is for the court to rule on the legal criteria which govern the question whether and in what circumstances public interest may justify disclosure. Within those criteria, a person should not be held to have acted unconscionably if their decision was reasonable’,^63^ although in the health context, significant deference is likely to be given by a court to the health professional and what their professional regulatory guidance advises about balancing interests in disclosure and maintaining confidentiality.

Regarding this second source of evidence (viz. regulatory guidance), Snelling and Quick demonstrate how the professional regulatory guidance for healthcare professionals, with respect to confidentiality and public interest disclosures, varies widely.^64^ They used three sources of benchmarking guidance on the issue of confidentiality: (i) the Department of Health’s NHS Code of Practice on confidentiality,^65^ (ii) its supplementary guidance on public interest disclosures,^66^ and (iii) the GMC guidance on confidentiality.^67^ From a close reading of these three sources, Snelling and Quick developed a framework of five questions as they concern public interest disclosure and areas of practical importance for health professionals: (i) Is public interest explained? (ii) Is the nature and level of harm to be avoided explained? (iii) Is intended beneficiary of disclosure explained? (iv) Is disclosure to prevent or detect serious crime explained? and (v) Is safeguarding explained?

Across the nine regulators examined,^68^ Snelling and Quick found that ‘…the quality of some health professional regulatory guidance is poor’^69^ and inconsistency reigns. Even though the guidance suggests that a common denominator for the justification for public interest disclosure is the *avoidance of harm* (both physical and psychological), its boundaries are porous. Some guidance, they note, fails to include the qualifier ‘serious’, which in their view sets the threshold for disclosure too low. Thus, it may not necessarily be the case, for example, that only ‘serious’ crimes such as murder, manslaughter, sexual assault, domestic violence, sexual abuse or neglect of children, and so on may meet the threshold; it also may be that less serious crimes such as theft or criminal trespass also are considered to justify disclosure in some of the health professions.

To take just their first question (viz. is public interest explained?) into deeper consideration, Snelling and Quick explore the GMC guidance’s explanation of public interest, as well as the Department of Health’s NHS Code of Practice on confidentiality, which defines the public interest as:

Exceptional circumstances that justify overruling the right of an individual to confidentiality in order to serve a broader societal interest. Decisions about the public interest are complex and must take account of both the potential harm that disclosure may cause *and* the interest of society in the continued provision of confidential health services.^70^

These two considerations—account of harm that disclosure may cause in the immediate sense and for the parties concerned, as well as the broader, more abstract impact that disclosure may cause on the continued provision of confidential health services—indicate to Snelling and Quick that regulators’ guidance explains public interest ‘fully’ only if it includes consideration of the additional benefit of maintaining confidential practice; in other words, the weighing must involve a balancing of public interests between disclosure and the maintenance of confidentiality. They note that evaluating where the public interest lies involves much more than weighing consequences in a specific case; additional weighting is required to account for the public interest in maintaining a confidential health service. This does not exactly address what public interest is and the robustness of its explanation, but it does go some way to teasing out how it is explained across the regulators’ guidance. And to that end, they find that only the GMC and General Chiropractic Council ‘fully’ explain the public interest in their guidance. The Nursing and Midwifery Council and Social Work England do not explain it at all in their guidance, and the five other regulators only ‘partially’ explain it, which as Snelling and Quick inform us, means the guidance has some discussion of the public interest, but does not mention the public interest in maintaining confidential health services in a general sense, in addition to the effects it might have on a specific patient.

The broader finding of inconsistency in regulatory guidance is bound to confound healthcare professionals and drive further uncertainty and confusion, and could well leave them to second-guess a regulator or the courts. Undoubtedly, this is troubling for professionals and the public alike because no one really knows where they stand until decisions are tested. This includes the key question for this article: is the scope of public interest in the medico-scientific context *necessarily* limited to preventing risk of serious harm to others and preventing or detecting serious crime?

What the above analysis indicates is that neither law nor guidance sufficiently pins down the public interest relative to how it impacts on the associated duty of confidence. Is this opportunity for expansion or grounds for caution? In law, we see an enduring trope of the public interest in upholding confidences as balanced against the public interest in disclosing confidential information. How does the public interest in upholding confidences sit alongside a public interest in science and a claim to a human right thereto? We know that a public interest in pushing for science cannot be treated as equivalent to a *right* to science, and any right would imply corresponding duties. So, how might we reconcile duties of confidence with duties to pursue science and reap its benefits? It may be time to revisit the trope of public interest versus public interest and consider a new framing of the values, rights, interests, and duties at stake. I begin to explore this in the following subsection.

### III.C. From Risk of Serious Harm to Promotion of Health?

To summarize the above discussion, the law will permit (restricted) disclosure of confidential information on a case-by-case basis where there are sufficient countervailing public interests favoring disclosure. In the medico-scientific context, the common law principles—and to some extent professional regulatory guidance—indicate that a *prima facie* case for disclosure will arise when the confidant reasonably assesses there to be a real risk of consequent danger to the public were the information *not* to be disclosed, and regardless of the confider’s consent or refusal for the information to be disclosed. Commentators have suggested that the risk of harm must be ‘real’ and not fanciful, and it must be a risk involving danger of physical or psychological harm.^71^ Moreover, the harm must be ‘serious’—minor harm (eg, the risk of passing a treatable or curable infection to one’s spouse) would not justify an override of both the specific disclosure and the general public interest in maintaining trust that confidants (be they health professionals or otherwise) will not disclose confidential information.

But must the scope of public interest be limited to only those situations? And in the case-by-case assessment, might we not question what counts as ‘harm’ in the specific context, including non-production of new medicines, health insights, vaccines, treatments, and health opportunities—what might be seen as harms by omission? Jurisprudence, academic commentary, legislation, regulatory guidance, and public opinion would suggest a broader scope than appears at first instance, and we ought to take heed from Lord Hailsham’s statement in *D v National Society for the Protection of Children* that ‘[t]he categories of public interest are never closed and must alter from time to time whether by restriction of extension as social conditions and social legislation develop.’^72^ We ought also, though, take heed from Simon Brown’s LJ recognition in *R v Department of Health Ex p. Source Informatics Ltd* of

… the importance of confining any public interest defence in this area of the law within strict limits—lest, as Gummow J put it at first instance in *Smith Kline and French Laboratories (Australia) Limited v Department of Community Services and Health* [1990] FSR 617, 663; it becomes ‘not so much a rule of law as an invitation to judicial idiosyncrasy by deciding each case on an ad hoc basis as to whether, on the facts overall, it is better to respect or to override the obligation of confidence’.^73^

Navigating the path between the flexibility of the common law to account for evolution of the public interest and the importance of curtailing judicial idiosyncrasy (without becoming speculative as to how each judge or court may operate) becomes the core challenge this section of the article tackles. I now to proceed to explore how the public interest has been interpreted in case law, academic commentary, legislation, regulatory guidance, and public opinion. What we begin to see is an openness to the idea that the public interest can cover a common interest concerning the promotion of collective welfare more broadly defined than previously considered.

#### III.C.1. Jurisprudence

Two medico-scientific cases are on point to suggest that the scope of public interest may stretch beyond cases of serious harm to others or serious crime. First, the case of *Lewis v Secretary of State for Health*^74^ considered whether certain documents relating to deceased patients, including their medical records, could be disclosed to a confidential inquiry co-sponsored by UK Government ministers looking into whether tissues removed from individuals who had worked in the nuclear industry had been lawfully removed and analyzed. The inquiry had not been set up under the Inquiries Act 2005 and, accordingly, had no statutory power to order disclosure of documents or to compel any person to provide it with documents. Under its general powers, the High Court authorized the disclosure and grounded this in a public interest basis. Foskett J held that:

…this is an appropriate case in which to hold that the public interest in disclosure of the material sought outweighs the other public interest, namely, that of maintaining the confidentiality of medical records and information, provided, of course, proper safeguards are put in place to ensure that no inappropriate information becomes public.^75^

The principal reason for this conclusion was:

…that there is plainly a public interest (and by that I mean not just ‘the interest of the public’) in determining what happened and why in connection with the very difficult and sensitive issue that arise from these matters. Those families that know broadly what happened are entitled to fuller answers to the questions raised if they wish to have them and there is a wider public interest in maintaining confidence in the NHS and the nuclear industry, a confidence that may be fortified either by the results of the investigation of The Inquiry or by the recommendations of The Inquiry if past practices are found to have been wanting and improvements are suggested.^76^

This would suggest the common law recognizes that disclosure of confidential information may be grounded in a public interest basis *beyond* preventing risk of harm to others and preventing or detecting serious crime; in this case, there was a public interest in determining whether tissues removed from individuals who had worked in the nuclear industry had been lawfully removed and analyzed, and at a larger scale, in helping contribute to a better understanding of how the workers died and which in turn could lead to improved confidence in both the health service and nuclear industry.

Importantly for the purposes of this article regarding the distinction between use of ‘confidential patient information’ for a ‘medical purpose’ (and use of section 251 support as the legal basis) and other kinds of confidential information that do not involve section 251, yet may nevertheless be of value in scientific research, Foskett J also held that while the information contained in the medical records was ‘confidential patient information’ within the meaning of section 251 of the National Health Service Act 2006,^77^ the purpose was *not* a ‘medical purpose’ within the meaning of the Health Service (Control of Patient Information) Regulations 2002 (‘the COPI Regulations’), in this case purposes of ‘the management of health care services’ in the sense that it would be subject to ‘audit, monitoring and analysis of patient care and treatment’ as per paragraph 5 of the Schedule to the COPI Regulations. This meant that the COPI Regulations and section 251 could not serve as a statutory gateway to lawfully disclose the confidential information. In the words of Foskett J:

It would, in my judgment, be wholly artificial and, perhaps, to some at least, an affront, to suggest that care for a patient ends at the moment of death; but equally it does have to be acknowledged that active care and treatment in the normally accepted sense of the expression does come to an end at that point. The answer of most people to the question ‘do you regard the removal of tissues from a deceased person for the purposes of analysis is part of the care and treatment of the patient?’ would surely be ‘no’. If that is so and it reflects the correct legal interpretation of the expression, the information obtained from this procedure – whether it be audited, monitored or analysed or a combination of all three – would not be being processed for a ‘medical purpose’ prescribed by the Regulations.^78^

Even if that were not the case, Foskett J continued, it could not be said that the inquiry itself, on proper analysis, was engaged in the ‘audit, monitoring and analysing of the provision made by the health service’ for the post-mortem analysis of tissues taken in the circumstances involved in the case:

… it does strain the normal meaning of the word ‘audit’ to embrace what The Inquiry will be setting out to do and, whilst I accept that The Inquiry is called upon to make recommendations, its principal focus is to discover what happened and why so that those affected, directly or indirectly, will have some explanation.^79^

He thus concluded that ‘endeavouring to bring the purposes of The Inquiry within the language of regulations drafted for a different purpose is a little difficult and does, in my view, involve a step too far.’^80^

Second, in the case of *R v Department of Health Ex p. Source Informatics Ltd*,^81^ the Court of Appeal considered an appeal against a declaration that the release of certain prescription information^82^ by pharmacists to pharmaceutical companies constituted a breach of confidence despite the protection of patients’ anonymity. Source Informatics contended that (i) information was only confidential to the patient if it could be identified with them and, in the circumstances, it could not; (ii) transfer of the material from one party to another was not misuse of it; and (iii) patients had not suffered any detriment. The Court of Appeal ruled that there was no breach of confidentiality as anonymity was protected. As *obiter dicta*, Simon Brown LJ also commented that confidential patient information used for the purposes of ‘thorough research and management’ could be acceptable:

For present purposes, I say no more than that, provided, as I understand to be the case, the use of such identifiable data is very strictly controlled, there appears no reason to doubt that this is acceptable—whether because it falls within the public interest defence or as is perhaps the preferable view, because the scope of the duty of confidentiality is circumscribed to accommodate it, it is not necessary to decide on this appeal.^83^

The qualifier ‘thorough’ research would suggest some degree of judicial openness to accepting restricted disclosure of confidential information, but the scope may well be limited to particular kinds of research (eg, public health emergency research) and under a number of strict conditions. I return to this additional consideration as well below.

#### III.C.2. Academic Commentary

Academic commentary would also suggest that the scope of public interest may stretch beyond cases of serious harm to others or serious crime. I have noted above that Phipps and colleagues observe that the public interest has broadened over time.^84^ Additionally, in their article, Snelling and Quick observe that ‘[t]he broad concept of public interest has been applied in three situations: (1) preventing serious harm to others, (2) preventing or detecting serious crime, or (3) enabling effective public health research’, yet they do not elaborate on the nature of this third situation, although (as elaborated below) the recent COPI Notices arguably serve as an example of this. Snelling and Quick do, however, reference an earlier article from Case, who in turn argues that the conceptualization of public interest in the common law—at least in England^85^—is *not* broad enough to accommodate health research. In her view, it appears to require an immediate risk of serious harm, at least based on case law developed through the early 2000s—which explains why it has instead been accommodated through statutory provisions that permit disclosure of ‘confidential patient information’ without consent for ‘medical purposes’, including medical research.^86^ It is to this important piece of legislation that I now turn.

#### III.C.3. Legislation

Specifically, the COPI Regulations were made by the Secretary of State using the powers conferred by section 60 of the Health and Social Care Act 2001. This section was repealed by the NHS (Consequential Provisions) Act 2006 and its statutory successor is section 251 of the National Health Service Act 2006. As the Health Research Authority (HRA)^87^ website states:

Section 251 was established as it was recognised that there were essential activities of the NHS, and important medical research, that required the use of confidential patient information where it was not possible to use anonymised information and obtaining consent was not practical.^88^

As Sorbie has observed, this legislation sought to address confusion around when it was acceptable for researchers to share confidential patient information in the public interest.^89^ Far from being a temporary solution as envisioned when first enacted, this statutory pathway for use of patient-identifiable information has endured for over 20 years, with no indication of it losing legislative or stakeholder support.^90^ This lends weight to Case’s argument that the conceptualization of public interest in common law was not seen as capacious enough to accommodate health research, and because consent or re-consent of patients was not seen as practicable for many forms of research, firm statutory footing was needed to enable confidential patient information sharing to cover situations beyond those concerning risk of serious harm to others or helping to prevent or detect serious crime. However, the scope covered by the statutory gateway is limited, even in scientific (or medical) research contexts, as will be seen.

The relevant subsections of section 251 of the NHS Act 2006 provide as follows (emphasis added):

Control of patient information

(1) The Secretary of State may by Regulations make such provision for and in connection with requiring or regulating the processing of prescribed patient information for medical purposes as he considers necessary or expedient—

(a) in the interests of improving patient care, *or*
(b) *in the public interest*.

…

(12) In this section ‘medical purposes’ means the purposes of any of —

(a) preventative medicine, medical diagnosis, *medical research*, the provision of care and treatment and the management of health and social care services, and
(b) informing individuals about their physical or mental health or condition, the diagnosis of their condition or their care and treatment.

Under the Act, ‘patient information’ is defined broadly as:

(a) information (however recorded) which relates to the physical or mental health or condition of an individual, to the diagnosis of his condition or to his care or treatment, and
(b) information (however recorded) which is to any extent derived, directly or indirectly, from such information, whether or not the identity of the individual in question is ascertainable from the information.^91^

And patient information is ‘confidential patient information’ where:

(a) the identity of the individual in question is ascertainable—
  (i) from that information, or
  (ii) from that information and other information which is in the possession of, or is likely to come into the possession of, the person processing that information, and
(b) that information was obtained or generated by a person who, in the circumstances, owed an obligation of confidence to that individual.^92^

The Regulations referenced in section 251 may make provision broadly under two headings: (i) for *requiring* prescribed communications of any nature which contain patient information to be disclosed by health service bodies or relevant social care bodies in prescribed circumstances, and (ii) for requiring or *authorizing* the disclosure or other processing of prescribed patient information, again in prescribed circumstances.

The public interest-based Regulations referred to in section 251 find their current form in the COPI Regulations. These Regulations explicitly set aside the common law duty of confidentiality: ‘Anything done by a person that is necessary for the purpose of processing patient information in accordance with these Regulations shall be taken to be lawfully done despite any obligation of confidence owed by that person in respect of it.’^93^

Regulation 5 of the COPI Regulations provides as follows:

Subject to regulation 7, confidential patient information may be processed for medical purposes in the circumstances set out in the Schedule to these Regulations provided that the processing has been approved –

(a) in the case of medical research, by both the Secretary of State and a research ethics committee, and
(b) in any other case, by the Secretary of State.

Based on what is set out in the COPI Regulations (including its Schedule), the HRA is responsible for appointing a committee for the purposes of giving advice to the Secretary of State and the HRA on the application of the COPI Regulations and the disclosure of confidential patient information, for both research and non-research medical purposes, without patient consent. The committee that has been appointed by the HRA for this purpose is known as the Confidentiality Advisory Group (CAG). CAG consists of upwards of 20 members and comprise a mixture of expert and lay members; meetings take place upwards of twice per month.^94^ The CAG acts as a safeguard through providing reassurance that section 251 applications are independently scrutinized by an impartial group before a final decision is taken.

It is clear that there is a public interest in promoting medical research to help improve health and wellbeing. For example, the Health and Social Care Act 2012, which largely applies only to England, makes it a duty of the Secretary of State to promote research on matters relevant to the health service.^95^ The same Act established the Health and Social Care Information Centre,^96^ which, *inter alia*, has duties to maintain standards, promote the core function of health services, and to develop a confidentiality code of practice. This builds on the statutory provision established by section 251 of the NHS Act 2006 that allows the Secretary of State to make provisions for the processing of patient medical data for medical purposes where (i) neither consent nor anonymization is possible or practicable, and (ii) when otherwise it would be a breach of confidence to use those data. The HRA has had responsibility to administer these powers since 2013. Applications and proposals for research uses, together with providing advice on draft regulations, are delegated to CAG, which advises decision makers—the HRA or the Secretary of State—whether applications to process confidential patient information without consent should be approved or not.

In terms of remit, the NHS Act 2006 and COPI Regulations have a number of inbuilt safeguards that the CAG considers as part of its assessment of applications from researchers to access confidential patient information.^97^ Among other things, this includes assurance that the activity must be in the public interest or in the interests of improving patient care, and that the public interest in the disclosure and potential benefits must, on balance, outweigh the breach of confidentiality.

The CAG also often considers whether patient groups or service users have been consulted to test the acceptability of the proposal to help identify the reasonable expectations of a patient on the proposed data use, and subsequently the public interest.

All of this, then, may be seen as the statutory creation of a public interest basis for disclosing confidential information without consent, albeit under a number of strict conditions and for a defined medical purpose. Undoubtedly, given the broad definition of ‘patient information’, ‘confidential patient information’, and the wide meaning attributed to ‘medical research’ under the NHS Act 2006, it is fair to assume that the section 251 statutory gateway is the primary legal basis for the vast majority of health research involving confidential information. However, as I have been keen to stress in this article, *not all forms of confidential information and research are captured by section 251*, and increasingly, health research and other kinds of scientific research involve a variety of data that do not constitute ‘confidential patient information’, including education, employment, financial, and pension data. The absence of a statutory gateway in these scenarios leaves the door open to a public interest grounded in the common law, as well as innovative policy formulation.

Before moving on, importantly for this article and the argument herein, it is also necessary to highlight Regulation 3 of the COPI Regulations. Regulation 3 both permits the processing of ‘confidential patient information’ and allows the Secretary of State to mandate processing for a range of public health purposes:

(a) diagnosing communicable diseases and other risks to public health;
(b) recognising trends in such diseases and risks;
(c) controlling and preventing the spread of such diseases and risks;
(d) monitoring and managing—
  (i) outbreaks of communicable disease;
  (ii) incidents of exposure to communicable disease;
  (iii) the delivery, efficacy and safety of immunisation programmes;
  (iv) adverse reactions to vaccines and medicines;
  (v) risks of infection acquired from food or the environment (including water supplies);
  (vi) the giving of information to persons about the diagnosis of communicable disease and risks of acquiring such disease.

With respect to the power to mandate processing, COPI Regulation 3(4) empowers the Secretary of State to require processing for these purposes by issuing a ‘notice’ to require a body or person specified in Regulation 3(3) to process that information for the purpose and time period specified in the notice. These are known as ‘COPI Notices’. In March 2020, at the beginning of the COVID-19 pandemic, the Secretary of State for Health and Care issued four COPI Notices, requiring, among other things, that NHS England, GPs, local authorities, combined authorities, and arm’s-length bodies of the Department of Health and Social Care process confidential patient information to support the Secretary of State’s response to COVID-19 (known as a ‘COVID-19 purpose’).^98^ A COVID-19 purpose included ‘research […] in relation to COVID-19’.^99^ These Notices were renewed repeatedly over the course of the pandemic and eventually expired in 2022, although one carried on through 2023.^100^ While the usual legal basis for many of the activities in a COPI Notice would have been Regulation 3 of the COPI Regulations, this is not the case for research, which is not explicitly mentioned in Regulation 3, although public health research may be seen to fall within its spirit.^101^ Much of the background discussion at the time the Notices were issued centered on the fact that the use of ‘confidential patient information’ for research in these circumstances was justified on the basis of overriding public interest, namely seeking insight regarding, and an evidence-based response to, the COVID-19 pandemic.

Following expiry of the COPI Notices, researchers and others relying on a COPI Notice and who needed to process confidential patient information for COVID-19 purposes would require an alternative legal basis for processing of confidential patient information thereafter, which could include obtaining patient consent, Regulation 3 support (presumably for non-research-related activities), or applying to the CAG to transition to Regulation 5 support (presumably for research-related activities, where applicable). Here, I would argue that in circumstances where a section 251 application (Regulation 5 support) is not feasible for one reason or another (eg, the confidential information sought is not ‘confidential patient information’), the COPI Notices themselves suggest that there may be an overriding public interest in using confidential information for research involving public health emergencies, even if the information is not ‘confidential patient information’ for which section 251 application (Regulation 5 support) is applicable. Thus, although the COPI Notices have expired as of the time of writing, the underlying principled justification for their enactment and support ought to be considered as still retaining value—something I consider in Sections 5 and 6.

What the foregoing analysis indicates is that far from being a temporary fix for concerns about the legal basis for using ‘confidential patient information’ without consent for secondary purposes such as research, legislation in England has endured for over two decades that charts a clear pathway for such use. It suggests three things. First, that the public interest can be codified in law and permit uses beyond those concerning risk of serious harm to others and risk of serious crime—in this case, a variety of forms of ‘medical research’. Specifically, this is public interest codified as a mechanism of section 251 support, where the disclosure and use can occur under a COPI Regulation and there is a mechanism for independent oversight and detailed consideration of the public interest; crucially, it is to be contrasted with the separate overriding (and non-statutory) public interest discussed in this article, which is (rightfully) more circumscribed because it is not accompanied by a similar oversight procedure. Second, though, it suggests that any future statutory developments similar to the COPI Regulations may curtail judicial discretion to develop the common law, particularly with regard to use or disclosure of ‘confidential patient information’ that might fall within scope of legislation. Yet, third, it also suggests that the basis for a broader expansion of the (non-statutory) overriding public interest is not human rights per se, including a human right to science, but rather a general recognition about the need for clear legal and policy positions that justify breaching confidentiality and waiving consent when confidential information other than ‘confidential patient information’ is used for research, and a more generalized principle that in the absence of such a legal or policy pathway, some forms of research that would be seen as being in the public interest would be wrongly thwarted.

#### III.C.4. Regulatory Guidance and Public Opinion

Finally, regulatory guidance and (limited) public opinion would also suggest the scope of the public interest basis may be broader than first appears. The Department of Health’s guidance from 2010, *Confidentiality: NHS Code of Practice—Supplementary Guidance: Public Interest Disclosures*, makes clear that there are no fixed categories of what a public interest basis for disclosure may be, and that decisions ‘must be made on a case-by-case basis’.^102^

The guidance lends weight to a view that serious ‘crime or harm to others’ are the primary categories of a public interest basis, yet the door is not closed to other categories, as the guidance also suggests a public interest justification for disclosure can be considered in situations where:

- Disclosure would be in the public interest; *and*
- The purpose of the disclosure cannot be achieved with anonymised information; *and*
- There is no statutory basis for disclosure; *and*
- Patient consent has not been given because:
  – It is not practicable to ask the patient(s) for consent because, for example, there are no up-to-date contact details for the patient, or the matter is urgent and the patient cannot be contacted; or
  – It would be inappropriate to ask the patient(s) because, for example, they lack the capacity to give consent, or they are suspect(s) who should not be informed that they are under criminal investigation; or
  – The patient(s) have been asked for consent and refused.^103^

While the first consideration is question-begging (what would be the specific public interest served for the disclosure?), the subsequent considerations leave open the door to situations that may concern something other than serious crime or harm to others. Indeed, the guidance goes on to state that:

There are clearly cases where disclosure of information may be in the public interest for a reason unrelated to serious harm or serious crime. The decision to disclose must take account of the likelihood of detriment (harm, distress or loss of privacy) to the individuals concerned, but a proportionate disclosure may be acceptable where there is clear benefit to the public. […] Similar considerations may apply to some research uses which do not affect the rights, freedoms or legitimate interests of individual patients.^104^

The guidance further goes on to note that because there is little case law in this area, it is recommended that advice be sought from the HRA’s CAG before making such a disclosure. A footnote in the guidance also states that in a research context, disclosure might occur ‘where a research ethics committee has advised that there is little or no risk of detriment to patients’,^105^ although it is questionable whether they always have the requisite competence to make such a determination.^106^

The British Medical Association (BMA) also has developed recent guidance in this area, albeit with a focus only on the medical (and patient information) context.^107^ It defines public interest as ‘the general welfare and rights of the public that are to be recognised, protected and advanced’,^108^ and states that a disclosure of confidential information can be in the ‘public interest’ if it is essential (i) to prevent, detect, or prosecute serious crime; (ii) to prevent a serious threat to public health or national security; or (iii) to protect individuals or society from serious harm.^109^ Clearly, then, the BMA guidance stresses the importance of a qualifier of ‘serious’ to limit the circumstances of breaching medical confidentiality, but does so in contexts broader than crime or harm to others, namely also considering the context of ‘serious threats’ to public health, although it seems to limit this to cases where an individual patient has a medical condition that puts others at risk or where a patient has a ‘serious communicable disease’, ^110^ rather than an epidemic or pandemic scenario in which entire populations carry or are at risk from an infectious disease—which, again, is understandable given the guidance is addressed to doctors engaged in a doctor-patient relationship where confidential patient information is disclosed. The guidance goes on to state that:

Disclosures in the public interest will generally be cases which relate to a single individual’s information. *Decisions about public interest disclosures must be made on a case by case basis*. The public interest test cannot be used to justify routine or ongoing disclosures.

Ultimately, the ‘public interest’ can only be determined by the courts.^111^

The Department of Health and BMA guidance thus suggests, indirectly, that judicial attitudes will have important but not necessarily full bearing on the development of the public interest. As judges can only rule on cases brought to them, judicial pronouncements alone will contribute only so much to the path of the public interest. Instead, expert committees such as CAG, as well as researchers and doctors themselves, not to mention Caldicott Guardians^112^ and legal counsel, have a key role to play in delineating the scope of the public interest. Here, we may also find that views of the public may well feed into (and should feed into) the policies developed, and decisions rendered, by these committees and professionals.

For example, it is worth noting that a 2018 Ipsos MORI poll of 2000 people, who were asked to give their views on sharing NHS patient address data for the purposes of investigate crime and immigration offences, found that a strong majority (71 per cent) supported the NHS sharing a patient’s address details with other government departments in order to trace an individual who has committed non-serious type of crime (eg, tax evasion, benefits fraud or theft), and with only 16 per cent opposed.^113^ This might indicate that the public has a wider view of the scope of a public interest basis for disclosure of confidential information than existing judicial pronouncements and regulatory guidance; it might also signal caution, though, that broad support in the abstract for a wider scope of the public interest does not always indicate broad support in practice, and the difficulty of translating preponderance/majoritarian views into a policy faithfully grounded in ‘the public interest’.^114^ This alone may be one reason why, in assessing on a case-by-case basis whether there is an overriding public interest to breach confidentiality and disclose information for a research purpose, particularly in the absence of an oversight body (beyond a research ethics committee) assessing such a case, the circumstances still ought to be limited to ‘serious’ situations where the public interest is manifest, such as research into public health emergencies.

To this end, the Department of Health’s and BMA’s guidance should be read alongside the NHS Constitution,^115^ which sets out a series of non-justiciable rights and responsibilities for patients and the NHS in England. Among other things, the Constitution commits that each patient has:

…the right to privacy and confidentiality and to expect the NHS to keep your confidential information safe and secure.

…the right to be informed about how your information is used.

…the right to request that your confidential information is not used beyond your own care and treatment and to have your objections considered, and where your wishes cannot be followed, to be told the reasons including the legal basis.

In turn, the NHS ‘pledges’:

to anonymise the information collected during the course of your treatment and use it to support research and improve care for others.

where identifiable information has to be used, to give you the chance to object wherever possible.

to inform you of research studies in which you may be eligible to participate.

This series of non-justiciable rights and responsibilities suggests that confidential (patient) information, particularly where identifiable, ought not to be used without patient knowledge and opportunity to object. A public interest override of this would be a tall order to meet the reasonable expectations of patients; many kinds of research are unlikely to meet those expectations. Yet other areas may well, particularly when we consider the Department of Health regulatory guidance that opens the door to ‘some research uses which do not affect the rights, freedoms or legitimate interests of individual patients’.^116^

In sum, then, jurisprudence, legislation, academic commentary, and regulatory guidance (alongside very limited public opinion) alike suggest that the scope of the public interest is not necessarily limited to preventing risk of harm to others and preventing or detecting serious crime, although the qualifier ‘serious’ continues to hold significant weight. Likewise, although not always overtly referenced, the notion of a risk or threat to wellbeing being imminent or immediate also seems to hold weight; this temporal element is something I return to below in thinking about the kinds of research scenarios that could be permissible on this basis of public interest buttressed by the human right to science. This means that broadening the scope to cover confidential information sharing for all kinds of research purposes, absent certain qualifiers, would be a step too far. It may be seen as too ‘indirect, cumulative, uncertain and non-immediate’ and:

… require some ingenuity to convince a court that the public interest defence can be used to breach the confidence of someone whose information is merely useful to others when combined with other information […] as opposed to someone who, alone, presents a specific and immediate danger to others.^117^

Yet, outside of section 251 scenarios involving confidential patient information, there does appear to be *some* scope for broadening the public interest to encompass *some* kinds of research—albeit seemingly largely confined to health research that has been considered by a research ethics committee and which makes use of confidential information for research regarding something like a public health emergency.

What none of these cognate domains have considered hitherto, however, is whether the basis for broadening of the scope of the public interest (again, outside the section 251 context) to cover research in scenarios such as public health emergencies may rest on the bolstering hook of human rights—and in particular human rights that have not per se been domesticated in UK law, unlike those in the ECHR. Thus, I turn now to consider the human right to science and the potential for its application in domestic jurisprudence as a buttress to an overriding public interest basis.

## IV. THE HUMAN RIGHT TO SCIENCE IN THE UK

The principle human rights framework that applies in the UK is drawn from the ECHR and its domestication through the HRA 1998. This means that the delineated rights from the ECHR are directly justiciable within its domestic courts. In relation to confidentiality law and the public interest, we have seen this play out in cases concerning breach of confidentiality—but even more so in cases concerning privacy interests, reflected in Article 8 ECHR, and a distinct cause of action, the emerging tort of misuse of private information^118^—both in terms of the limitation to the right to privacy afforded in Article 8(2) ECHR^119^ and in terms of a public interest in freedom of expression, reflected in Article 10 ECHR. Both Article 8(2) and Article 10 ECHR can be seen as ‘checks’ on privacy interests that are akin to the exception represented by the public interest in common law. Indeed, it is worth noting English courts’ growing recognition of the importance of ECHR-delineated human rights, which date from 1950—*even before* the Convention was domesticated in UK law under the HRA 1998. For analogical purposes, it is worth exploring how human rights analysis fits within the misuse of private information tort.

### IV.A. Human Rights Analysis within Misuse of Private Information and Breach of Confidence Claims

The public interest also serves as a defense in claims for misuse of private information, where human rights feature in the analysis much more pronouncedly than they do in breach of confidence claims. Here, courts generally adopt a two-stage process addressing two broad issues: (i) whether Article 8 ECHR is engaged at all, which depends in turn on whether the claimant had a ‘reasonable expectation of privacy’ in respect of the relevant information; (ii) if Article 8 ECHR is engaged, whether the defendant’s interference with the claimant’s right to privacy was justified by other relevant considerations, such as the defendant’s own rights, the rights of others, and/or the public interest. In some of these cases, particularly those involving publication (or threatened publication) of private information by a media organization or person, Article 8 is balanced against Article 10 (right to freedom of expression).

The UK Supreme Court case of *Bloomberg LP v ZXC*^120^ has affirmed that for the first stage, whether there is a reasonable expectation of privacy is an objective question. With respect to the second stage, there is no test or formula to determine which interest should prevail in a given case. A balancing exercise is carried out in each instance to determine which interest should prevail. As Lord Steyn noted in *Re S (A Child)*, proportionality is the key factor in examining the precise scope of confidentiality (or privacy) and freedom of expression rights being claimed:

First, neither article [of the ECHR] has *as such* precedence over the other. Secondly, where the values under the two articles are in conflict, an intense focus on the comparative importance of the specific rights being claimed in the individual case is necessary. Thirdly, the justifications for interfering with or restricting each right must be taken into account. Finally, the proportionality test must be applied to each.^121^

And, as noted by the Court of Appeal in *Associated Newspapers Ltd v Prince of Wales*,^122^ which involved a breach of confidence claim, the key question is whether it is in the public interest that the duty of confidence should be breached (or, analogously, that private information be used and disclosed), and given this means overriding a private law right, factors of real weight and importance must be present:

There is an important public interest in the observance of duties of confidence. […] Before the Human Rights Act came into force the circumstances in which the public interest in publication overrode a duty of confidence were very limited. […] Today the test is different. It is whether a fetter of the right of freedom of expression is, in the particular circumstances, ‘necessary in a democratic society’. It is a test of proportionality. But a significant element to be weighed in the balance is *the importance in a democratic society of upholding duties of confidence that are created between individuals*. It is not enough to justify publication that the information in question is a matter of public interest. […].

For these reasons, the test to be applied when considering whether it is necessary to restrict freedom of expression in order to prevent disclosure of information received in confidence is not simply whether the information is a matter of public interest but *whether, in all the circumstances, it is in the public interest that the duty of confidence should be breached*. The court will need to consider whether, having regard to the nature of the information and all the relevant circumstances, it is legitimate for the owner of the information to seek to keep it confidential or whether it is in the public interest that the information should be made public.^123^

Article 10 ECHR provides that everyone has the right to freedom of expression, but also provides that this freedom may be subject to such restrictions as are prescribed by law *and* are necessary in a democratic society for (among other things) protecting the rights of others and/or preventing the disclosure of information received in confidence. Even before the ECHR had been incorporated into domestic law, Lord Goff in the *Spycatcher* case recognized the importance of balancing human rights against disclosure of information received in confidence:

… I can see no inconsistency between English law on this subject and article 10 of the European Convention on Human Rights. […] The exercise of the right to freedom of expression under article 10 may be subject to restrictions (as are prescribed by law and are necessary in a democratic society) in relation to certain prescribed matters, which include ‘the interests of national security’ and ‘preventing the disclosure of information received in confidence’. It is established in the jurisprudence of the European Court of Human Rights that the word ‘necessary’ in this context implies the existence of a pressing social need, and that interference with freedom of expression should be no more than is proportionate to the legitimate aim pursued. I have no reason to believe that English law, as applied in the courts, leads to any different conclusion.^124^

In English law, prescription by law is to be found in the established common law principles of confidentiality. This means that in any case, a claimant must bring the claim within those principles. As Phipps and colleagues note, in so far as the claim is based on the equitable doctrine of confidentiality (rather than, say, a contractual basis), it involves the test of the reasonable recipient’s conscience.^125^ Moreover, ‘[t]he principles include recognition that in some cases a duty of confidentiality may be negated or qualified by public interest. Proportionality in this context is an aspect of the relevant principles rather than a separate principle.’^126^

From this analogy to the tort of misuse of private information analysis, a key question arises: may one extend the ECHR-driven analysis to a human right to science, which, even if not incorporated into domestic law per se, would *prima facie* permit a court or other competent body to consider whether another human right—namely the promotion of scientific progress and its application—would override a duty of (or right to) confidentiality in a particular set of circumstances—and if so, what extra weight (if any) is the human rights ‘gloss’ adding to the public interest basis? Does the human right to science serve as a basis for a conflict of rights argument,^127^ as weighed against a common law duty of/right to confidentiality, or is it rather more of a juridical ‘buttress’ to a public interest basis for confidential information disclosures?

### IV.B. What Does the Human Right to Science Entail?

As noted above in the Introduction, one right enshrined in the UDHR is the right of everyone to ‘share in scientific advancement and its benefits’ (Article 27). Scholars consider the right to have at least a dual-pronged nature: the promotion of the beneficial effects of science, as well as the protection against its adverse effects.^128^ In 1966, this right was incorporated into the ICESCR, a binding treaty that refers explicitly to the UDHR and represents an international endeavor to give legal force to the rights, and to which, to date, 171 countries have voluntarily agreed to be bound. This includes the UK, but the UK is not a signatory to the Optional Protocol to the ICESCR, which means it does not agree to recognize the competence of the Committee on Economic, Social and Cultural Rights to consider complaints from individuals or groups who claim their rights under the ICESCR have been violated. Unlike most other human rights, the right to science has never been legally defined and many countries, including the UK, have seemingly forgotten about its existence, much less its implementation.^129^ It should be added that the ECHR itself does not contain a provision on the right to science, and nor is it provided in the Council of Europe’s European Social Charter. In itself, this suggests that a ‘right to science’ may sit uneasily with many governments, particularly regarding what it would mean in practice and the reasonable limits one ought to set around the various entitlements and responsibilities that attach to it.

The principal right to science is located in Article 15(1)(b) ICESCR, which denotes the right of everyone to enjoy the benefits of scientific progress and its applications. The article also expounds on the obligations of States parties to take steps for the conservation, development, and diffusion of science (Art. 15(2)), to respect the freedom indispensable for scientific research (Art. 15(3)), and to promote international contacts and cooperation in the scientific field (Art. 15(4)). Yotova and Knoppers argue that the human right to science has two main aspects: the right to access scientific knowledge and information and, second, the right to benefit from scientific applications.^130^ It is the first aspect that particularly speaks to the value of data sharing; after all, access to scientific knowledge is necessarily contingent on access to underlying data to drive analysis and understanding, leading to information and ultimately to knowledge.^131^ They also observe that this right comes to include, ‘in its core content, a right to access scientific information, including data and scientific publications, to be enabled by states’.^132^

### IV.C. A Conflict of Rights?

In her 2012 report to the Human Rights Council, the Special Rapporteur in the field of cultural rights, Farida Shaheed, noted that the ‘scope, normative content and obligations of the State under this right […] remain underdeveloped while scientific innovations are changing human existence in ways that were inconceivable a few decades ago’.^133^ The Special Rapporteur emphasized the link between the rights to science and the right to participate freely in the cultural life of the community, as well as with the freedom of expression. She remarked on both its intrinsic value and instrumental value as a prerequisite for the realization of a number of other human rights (eg, rights to health, water, housing, and education; right to development; right to a clean and healthy environment). She elucidated that:

The terms ‘benefits’ of science and ‘scientific progress’ convey the idea of a positive impact on the well-being of people and the realization of their human rights. The ‘benefits’ of science encompass not only scientific results and outcomes but also the scientific process, its methodologies and tools.^134^

The Special Rapporteur stated that the human right to science may, as with most other rights, be subjected to limitations, in accordance with relevant international standards:

…such limitations must pursue a legitimate aim, be compatible with the nature of this right and be strictly necessary for the promotion of general welfare in a democratic society, in accordance with article 4 of the Covenant on Economic, Social and Cultural Rights.^135^ Any limitations must be proportionate: the least restrictive measures must be taken when several types of limitations may be imposed. Furthermore, existing international human rights standards on limitations that can or cannot be legitimately imposed on rights intrinsically linked to the right to science, such as the rights to freedom of opinion and expression, to information and to association, taken into consideration.^136^

Only limited mention was made, however, regarding limitations that might be applied to using confidential (or otherwise private) information to realize the human right to science, much less how this right might conflict with other rights or duties, including privacy and confidentiality. The Special Rapporteur noted that:

Conducting research in a socially responsible manner in accordance with ethical standards is emphasized in article 14 of the Universal Declaration on the Human Genome and Human Rights. Rights and freedoms that may be most threatened by the conduct of scientific research, especially those involving exposure or contact and social science research eliciting personal data, are the rights to physical and intellectual integrity, liberty and security, to privacy, and to seek, receive and impart information.^137^

No mention of limitations in this context was made either in a two-day seminar held in 2013 and convened by the Office of the United Nations High Commissioner for Human Rights (OHCHR) beyond a general recognition that scientific freedom is ‘not absolute and must be enjoyed responsibly’.^138^ No mention is made in secondary literature either, suggesting limited guidance to date on how to reconcile any conflict between the human right to science and, say, the right to privacy or a broader (common law) right to confidentiality in one’s personal health information. Indeed, this raises questions about how respecting a ‘right’ to confidentiality might in turn inhibit the right to science (eg, incomplete data can adversely impact data quality and scientific progress), and how to reconcile a civil right with a socio-cultural right, particularly when all human rights are seen as universal, inalienable, indivisible, interdependent, and interrelated.

In 2020, the United Nations Committee on Economic, Social and Cultural Rights (CESCR) published General Comment No. 25 on Science and Economic, Social and Cultural Rights, which provides much-needed interpretative guidance on the right to science and a basis for measuring and monitoring implementation of the right. The General Comment noted that ‘science’ ought to be interpreted broadly (eg, every possible branch of scientific research), and scientific ‘applications’ ought to be interpreted broadly and include the technology deriving from scientific knowledge, such as medical applications, industrial or agricultural applications, and information and communications technology. Likewise, it was stated that:

The term ‘benefits’ refers first to the material results of the applications of scientific research, such as vaccinations, fertilizers, technological instruments and the like. Secondly, benefits refer to the scientific knowledge and information directly deriving from scientific activity, as science provides benefits through the development and dissemination of the knowledge itself. Lastly, benefits refer also to the role of science in forming critical and responsible citizens who are able to participate fully in a democratic society.^139^

General Comment No. 25 does, however, explicitly recognize the limitations of the human right to science and in particular as it concerns the use of potentially confidential information: ‘Acceptability [of the right] implies also that scientific research has to incorporate ethical standards in order to ensure its integrity and the respect of human dignity, […] Some of these standards are that […] privacy and confidentiality should be respected […].’^140^ Similar to the Special Rapporteur’s report, General Comment No. 25 also observes that:

…limitations on the right must respect the requirements of article 4 of the Covenant: first, limitations have to be determined by law; second, they must promote ‘the general welfare in a democratic society’; and third, any restriction must be compatible with the nature of the right restricted. As understood by the Committee, this implies that limitations must respect the minimum core obligations of the right, and must be proportionate to the aim pursued. This means that where there are several means reasonably capable of achieving the legitimate aim of the limitation, the one that is least restrictive to economic, social and cultural rights must be selected, and the burdens imposed on the enjoyment of the right should not outweigh the benefits of the limitation.^141^

While General Comment No. 25 does not go into any detail about how limitations may be applied in the context of protecting the confidentiality of certain information, and does not mention how science and scientific progress significantly depends on sharing of data—including at times identifiable data that may be private or confidential—it does provide helpful clarification regarding the contours of the human right to science and how any limitations on the right must be carefully considered. It also lends weight to an argument that a public interest basis to lawfully share confidential information would necessitate a balancing exercise similar to that conducted in cases regarding the right to privacy under Article 8 ECHR and freedom of expression under Article 10 ECHR. Indeed, the analogy to Article 10 ECHR is apposite given that Yotova and Knoppers observe that ‘in Europe, with its many scientifically and technologically developed states, the right to benefit from science is understood as a form of collective freedom of expression rather than as a positive right’.^142^

The limiting factor here, however, is the English courts’ hesitation to give effect to social and cultural rights of the kind delineated in the ICESCR, given that the treaty, although signed and ratified by the UK, has not been domesticated in law as has the ECHR. Moreover, we must recall that the right *to* science entails a right of everyone to share in its benefits; this ought to be distinguished from a right *of* science, which would include the right of scientists to engage in their research free from interference (read as a broader version of freedom of expression under Article 10 ECHR).^143^ This means that neither the ICESCR’s general principles nor its substantive provisions can be enforced by domestic courts. Thus, even though General Comment No. 25 stresses that the human right to science ‘is enforceable and is therefore also justiciable’,^144^ realistically, this has not come to fruition in the UK. Nor, it should be added, in many other countries; evidence suggests very limited relevant domestic and international case law offering judicial insight into the right.^145^

This position is reflected in the UK’s seventh periodic report under the ICESCR (2022), submitted by the Ministry of Justice.^146^ The report does not mention the human right to science at all, and notes only in general terms that ‘The UK has implemented a combination of policies and legislation to give effect to the UN human rights treaties it has ratified’,^147^ while failing to indicate that because the ICESCR has not been domesticated in law, the human right to science is not per se enforceable or justiciable. The UK Government reiterated, too, that it does not intend to sign the Optional Protocol as ‘the benefits of the communication procedure remain unclear, especially for the applicant’ and believes that ‘effective domestic laws already exist where individuals can seek enforceable remedies if their rights have been breached. It is possible for an individual to challenge any government decision in the domestic courts if their rights have been breached.’^148^ This continues a trend that can traced back to the UK’s first report from 1993, in which the Government justified the absence of incorporating the right to science in domestic legislation on the grounds that ‘[n]o legislation or other government measures have been taken, or are considered necessary, to guarantee that right’,^149^ suggesting that ‘domestic law silences do not necessarily imply negation of the right’.^150^ Yotova and Knoppers add, importantly, that: ‘It should be borne in mind that a number of domestic legal systems incorporate customary international law automatically, including, arguably, the right to benefit from science, and thus do not require express legislation to give it effect.’^151^

### IV.D. The Human Right to Science as a Juridical Buttress to the Public Interest

All this, then, does not foreclose a domestic court from giving due regard to the human right to science as a public interest basis in a breach of confidence claim, but it does suggest the weight a court would afford it would be less than a right emanating from the ECHR and HRA 1998, thus undermining the strength of a conflict of rights claim as between an arguably non-justiciable human right to science and a civil right and common law right (and duty) of confidentiality. It also suggests that the human right to science may be under-implemented in the UK and thus unduly thwart sharing of data to foster scientific and technological development, affecting an appropriate balance between data sharing and respect for confidentiality. Yet there is another limiting step, as Yotova and Knoppers observe that:

The key challenge to the realization of the right to benefit from science is that, as indicated by the reports of the states parties, most scientific research and the generated data are privately funded. This makes it much more difficult for states to enable access to it, given that human rights do not impose obligations directly on private parties absent explicit domestic regulation to this effect and that such measures are scarce in state practice so far.^152^

Given that in the UK, human rights tend to be seen as having vertical effect, binding the state (as obligation-holder) to the individual (as beneficiary), and only indirect horizontal effect (ie, not binding individuals and private actors, but enabling an individual claimant to bring a rights violation claim against the state rather than the responsible non-state private actor), the value of the human right to science as a buttress to a public interest basis may be constrained from a lawfulness perspective. Namely, while the HRA 1998-recognized human rights to privacy and freedom of expression are seen as rights of indirect horizonal effect in English law, it is far from clear that the non-domesticated ICESCR’s human right to science would be treated the same. And, from a social license perspective, this constraint may be even more pronounced in contexts where commercial actors seek access to confidential information: empirical research indicates greater reluctance and skepticism among the general public for their data to be used for commercial purposes than for publicly funded research that has a more explicit public purpose, such as a research involving a public health emergency.^153^

At most, given the dominant approach of vertical effect and indirect horizontal effect, one would expect courts to acknowledge the UK’s obligation to give effect to the ICESCR’s rights, including for the full realization of the right to participate in and to enjoy the benefits of scientific progress and its applications, but that this would not mean an obligation to consider the impact of the law of confidentiality on the realization of any of the Covenant’s rights in the same way as it might in a consideration of its impact on an ECHR right such as freedom of expression. This would especially be the case where underlying scientific research is privately funded, as it remains an open question whether domestic courts would afford any difference in the interpretation and application of the right between state-funded and privately funded research. Instead, a more general and active recognition of the human right to science—giving it due regard—may provide a kind of buttress to a public interest analysis for assessing the circumstances in which there may be lawful sharing of confidential information. In this article’s context, this would rest particularly on a putative claim by scientists that having defined access to confidential information is necessary and proportionate to the needs for researchers to conduct science, achieve scientific progress, and ultimately disseminate scientific benefits to the public. In the following section, I proceed to consider how such a claim might play out through two hypothetical scenarios that could invoke the human right to science as a buttress to a public interest basis for disclosure of confidential information.

## V. TWO HYPOTHETICAL SCENARIOS INVOLVING THE PUBLIC INTEREST

In this section, I consider two hypothetical scenarios, sketched at a relatively impressionistic level, in which a research team wishes to access various kinds of confidential information relating to a significant number of individuals, and a query arises as to whether a public interest basis may be considered lawful in the circumstances, particularly when grounded in the human right to science.

### V.A. The Hypothetical Scenarios

***Hypothetical scenario 1***  *Dr Anil Singh has been undertaking a publicly funded study on the incidence of COVID-19 diagnosis, hospitalization, and death in healthcare workers across England, seeking to establish whether there are differences in these COVID-19 outcomes between ethnic minority workers compared to their White colleagues. This involves undertaking a retrospective cohort study using data from multiple linked electronic databases, including identifiable NHS human resource, health regulator data, and various NHS datasets. Dr Singh and his team have assessed that it is either impossible or impractical to gain consent from potential research participants The study is not collecting personal information directly from any individual healthcare workers; instead, identifiable information of approximately 1 million healthcare workers will be provided by regulatory bodies such as the GMC, the Nursing and Midwifery Council, and the General Pharmaceutical Council. Further, the NHS employee records service (known as the Electronic Staff Record, or ESR) will supply employment information about healthcare workers. In order to link the healthcare worker cohort to healthcare outcomes data (to assess COVID-19 outcomes), identifiable data will be shared within NHS England. This identifiable data will be received and pseudonymised by a special health authority that specializes in health informatics, and the pseudonymised data will flow to a trusted research environment (TRE), where it will be encrypted (rendering it anonymous) and stored for analysis. The duty of confidentiality is relevant when considering the sources of data used for analysis. The duty specific to the healthcare context is engaged through use of both primary and secondary care data (which would constitute ‘confidential patient information’). The duty of confidentiality is also engaged in the use of employment data (which is not ‘confidential patient information’), because as part of the contractual employment relationship, an employer (eg, the NHS) has a duty to keep an employee’s data confidential. As such, the participants in the study have a reasonable expectation that their information, be it health or employment-related, will remain confidential and not be used in ways that deviate from their reasonable expectations, unless there is a specific legal basis that lifts the duty of confidentiality. The COPI Regulations cover use of confidential patient information on the basis of the COPI Notices (up until June 2022) and thereafter on the basis of section 251 support and CAG approval. To that end, Dr Singh has applied for and received CAG approval for use of the confidential patient information. However, the COPI Regulations do not apply to this study insofar as workforce employment data (ie, non-‘confidential patient information’) is concerned and thus Dr Singh seeks an appropriate legal basis for use of this information. Dr Singh has obtained a favorable opinion from an NHS research ethics committee for his study.*

 

***Hypothetical scenario 2***  *The Sheffield Teaching Hospitals NHS Foundation Trust wishes to establish a research database aiming to understand the relationship between child health issues and educational attainment levels within the Sheffield locality. The sample to be included within the database will be all individuals within the Sheffield locality who were born between 01 January 1996 and 31 December 2022. It is proposed that routinely collected data from the following sources will be linked to create the research database:*

*1. Primary care data from all 76 GP practices across the Sheffield region;*

*2. Secondary care inpatient, outpatient, and emergency care data from Sheffield Teaching Hospitals NHS Foundation Trust;*

*3. Community care data from Sheffield Health and Social Care NHS Foundation Trust; and*

*4. School education data from Sheffield City Council, which receive this information directly from the schools, as well as linkage to educational data held by the Department for Education.*

*Confidential patient information will be provided by all healthcare providers participating in the study, in order to facilitate linkage across datasets. The school education data source detailed above would not fall within the definition of ‘confidential patient information’ as defined in s251(11) of the NHS Act 2006, but does contain individually identifiable information (eg, name, gender, address, date of birth, school, attendance, GCSE results), and the Foundation Trust is keen to link these data with the health data. The duty of confidentiality is engaged in the use of education data because as part of the school/teacher-pupil relationship, a school, teacher (and council) has a duty to keep the pupil’s education data confidential, subject only to disclosure, as necessary, to the pupil’s parent(s) or legal guardian(s). As such, the participants in the proposed research database have a reasonable expectation that their information, be it health or education-related, will remain confidential and not be used in ways that deviate from their reasonable expectations, unless there is a specific legal basis that lifts the duty of confidentiality. Given the large population-wide study, the Foundation Trust have assessed that it is either impossible or impractical to gain consent. The team is considering how best to proceed concerning various approvals, including NHS research ethics committee approval and section 251 approval through the Confidentiality Advisory Group to obtain support for disclosure of the confidential patient information. The team is also considering how to obtain confidential information concerning the school education data without consent (from adolescents and/or parents), namely by way of a public interest justification.*

In hypothetical scenario 1, confidential information is sought for a clear research-related purpose: assessing potentially different care outcomes stratified by ethnicity during a public health emergency, specifically a pandemic that has caused the deaths of millions of people globally and several hundred thousand people in the UK, including a significant number of healthcare workers. Moreover, insofar as non-confidential patient information is involved, the statutory gateway afforded by section 251 of the NHS Act 2006 and the COPI Regulations is not available for use, meaning that the research team will need to rely on another legal basis to access and use the relevant confidential information. The question thus arises: can the legal basis be the public interest, and if so, how might it be supported (if at all) by the human right to science claim?

In hypothetical scenario 2, we are outside the realm of a public health emergency, but still firmly within the realm of scientific research and a bona fide study with bona fide researchers. While CAG may provide section 251 support and approval insofar as it relates to non-consented disclosure and use of confidential patient information, they may likely do so provided certain conditions are fulfilled. This may include a requirement that the team in charge of establishing the research database ensure that appropriate security arrangements are in place; requiring all staff at Sheffield City Council who are involved in processing information for this study should have successfully completed local security awareness training before processing any information; confirmation that the study has received a favorable opinion from an NHS research ethics committee; and support from NHS England’s data advisory team that suitable information governance processes are in place, including by way of a Data Security and Protection Toolkit (DSPT) submission.^154^ Similar to hypothetical scenario 1, here, too, a question arises regarding how the confidential information may be accessed, if at all, on grounds other than consent—namely through the (overriding) public interest as a legal basis.

### V.B. Evaluative Frameworks from Law and Ethics

Two main evaluative frameworks may be applied to assist the assessment, one drawn from law and another from ethics. The value of these evaluative frameworks to the question I am addressing in this article is that, together, a kind of ‘hybrid’ approach where a legal framework is complemented by an ethics approach may serve best to work through the reasoning in a given scenario to assess whether there is a lawful (and ethical) basis for disclosure of confidential information. Not only would this help ensure confidential information is disclosed within the bounds of the law, it would also help ensure that doing so is ethically justifiable and in accordance with societal views and expectations.

The first evaluative framework may be sourced from the case law and professional guidance referenced above, including case law concerning the ECHR in which certain conditions have to be met before lawful exceptions to a human right would be satisfied. This would require one to weigh up in a given scenario three considerations:

(1) whether such disclosure (and use) is seen as a proportionate action, weighed between the public interest in maintaining confidentiality and the benefit to the public to address the particular research question; and
(2) whether a proportionate disclosure may be acceptable where there is clear benefit to the public.

To the first question, this should entail consideration of whether disclosure and use can be seen as genuinely in pursuit of a research purpose (involving a scientific endeavor), and, taking all relevant circumstances into account, whether it is seen as necessary in a democratic society for those ends. To the second question, the recent guidance from the National Data Guardian, on the evaluation of public benefit when health and adult social care data about patients or service users are processed without their consent for purposes beyond individual care, is of particular use.^155^ The guidance defines public benefit in terms similar to public interest and the discussion below concerning ‘net interest’:

Public benefit means that there should be some ‘net good’ accruing to the public; it has both a benefit aspect and a public aspect. The benefit aspect requires the achievement of good, not outweighed by any associated risk. Good is interpreted in a broad and flexible manner and can be direct, indirect, immediate or long-term. Benefit needs to be identifiable, even if it cannot be immediately quantified or measured. The public aspect requires demonstrable benefit to accrue to the public, or a section of the public.^156^

As the guidance notes, there are two aspects to public benefit evaluations: the public aspect and the benefit aspect. For the former, ‘The project or initiative that is applying to use data must be motivated by an intention to benefit the public, or a section of the public.’^157^ For the latter:

Data recipients should be prepared to demonstrate what public benefit the data use is delivering. This should happen at such intervals as specified by the organisation providing access to the data. It should also be in a form that can be readily shared with the public, for example, on a data uses register.^158^

Moreover, there is a third consideration at play in assessing whether confidential information may be accessed on a public interest basis:

(3) a legal evaluative framework would involve a consideration of whether the affected individuals have a reasonable expectation of confidentiality in respect of the relevant information,^159^ and whether the research team’s interference with these individuals’ confidentiality interests is justified by other relevant considerations, in particular a public interest in such a research use, buttressed by a human right to science.

I would argue that a public health emergency, which can be defined as ‘an occurrence or imminent threat of widespread or severe damage, injury, or loss of life or property resulting from a natural phenomenon or human act’,^160^ would be considered a public interest that could constitute a valid legal basis for disclosure and use of confidential information. It would clearly meet a common understanding of the public interest and reflects a ‘net interest’ in which all members of the public have in common, viz. combatting a live or imminent public health threat where there is risk of widespread or severe harm (in other words, a serious health threat to the general population). By ‘net interest’, I mean (taking up Barry’s account of the public interest^161^) that it may well be the case that people in England can have different interests in respect of a public health emergency and research on it, insofar as they simultaneously occupy different roles or capacities in relation to it. Each person may balance their various interests differently according to their preferences, but, on net, they reflect a collective interest for *all* members of society rather than one which attaches only to particular individuals or groups. Other kinds of serious health threats to the general population, which may or may not be categorized as a ‘public health emergency’, such as climate change, air pollution, cancer, and mental health crises, may be less likely to meet the public interest threshold in this context given, among other reasons, the difficulty with defining the specific research problem and establishing the imminence or immediacy of the threat of widespread or severe harm to human health, although I acknowledge there may be strong counter-arguments here.

The second evaluative framework is drawn from ethics and may help inform legal reasoning. Specifically, one may consider applying to the scenarios Schaefer and colleagues’ definition of public interest, which may serve as an evaluative ethical framework. They apply their definition to considerations of consent waivers in the context of biomedical research, but it may be seen to have purchase in the confidentiality context, too. They define public interest as ‘substantial expected advancement of the health-related interests of members of a group whose interests are, or should be, of particular concern to the society in question.’^162^ They make their definition relative to groups whose interests are of particular concern to advance via the research, such as a rare disease community, and groups who traditionally have been marginalized and whose interests have been (or continue to be) unjustly ignored by society at large. In the context of a public health emergency, *all* members of a community would have a common interest in seeing a response to address it, be it a coordinated response from the state or responses from various actors, including researchers (which may involve support from the state). This definition enables us to consider the interests at stake, the value of the research, and whether the threshold is met such that a public interest disclosure can, in principle, be made lawfully. Schaefer and colleagues also offer a helpful set of criteria (which, they emphasize, can only provide ‘non-exhaustive categories which *tend towards* contribution to the public interest’,^163^ due to the shapeshifting nature of the public interest) that could assist in a systematic evaluation of the public interest. These criteria include addressing a health priority; scientific robustness; open access of publications and datasets as purported research outputs; non-patentability/copyright of findings to keep them in the public domain; and translatability (ie, research whose results have direct, measurable relevance to practice or policy). Again, I would argue that many types of research in the context of a public health emergency would very likely fulfil these criteria.

Presumably, then, applying both sets of evaluative frameworks to the two hypothetical scenarios, Dr Singh and the research team would make an argument that there is a public interest basis in having the confidants make a limited, carefully controlled disclosure of the confidential information. Here, the research team would want to make an argument that disclosure is seen as a proportionate action weighed between the public interest in maintaining confidentiality and the benefit to the public to address the particular research issue (COVID-19). Moreover, they would want to advance an argument that, similar to an ECHR Article 8 versus Article 10 exercise, the human right to science obliges a balancing exercise between a right to confidentiality and a right to science, including access to information for research that is in the public interest. Alternatively, they may wish to advance an argument more akin to an Article 8(1) versus Article 8(2) exercise, namely that the human right to science serves as a buttress to the ‘protection of health or morals, or […] the protection of the rights and freedoms of others’ such that identifiable information in the particular circumstance should be disclosed. In this scenario, there would seem to be a stronger argument for disclosing the confidential information on the grounds an overriding public interest, with the human right to science helping support that legal basis.

Conversely, under hypothetical scenario 2, despite there being a legitimate research question and aim to establish a research database using the datasets requested, obtaining individual-level confidential school education data on a public interest basis, supported by a human right to science claim, is more tenuous. It is not at all clear that the school education data must be obtained at an individually identifying level and that the research team themselves ought to perform the data linkage rather than an independent, accredited unit (including in a trusted research environment/secure data environment or data safe haven). Likewise, it is arguable that the disclosure of this information in this context would not constitute a proportionate action such that the benefit to the public in establishing this research database outweighs the public interest in maintaining confidentiality in children’s’ and adolescent’s school records. Given the non-emergency scenario here, the broad, open-ended nature of the longitudinal study, and the diminished sense of a clear benefit to the public, it is unlikely that any proportionate disclosure of confidential information here would be viewed as acceptable in the absence of consent. Indeed, one could surmise that both pupils and their parents/legal guardians have a reasonable expectation of confidentiality in respect of the relevant information and that disclosure of the information for this research database would not run in accordance with that reasonable expectation. The human right to science would not serve as a juridical buttress here in the absence of an interest in which all members of the public have in common, such as a live or imminent public health threat where there is risk of widespread or severe harm.

Invariably, any scenario that is put to both sets of evaluative frameworks entails an assessment of the positive and negative aspects of the proposed research endeavor (which may well also entail a consideration of the value or social desirability of each endeavor, in addition to merit of the science), as well as a weighing of the trade-offs between different elements of the common good, including confidentiality, privacy, trust, justice, and population health. Whether buttressed by the human right to science or not, any overriding of the public interest in keeping identifiable health data confidential will necessitate careful consideration and, as Ballantyne and Schaefer suggest, ‘a wide population scope interpretation of public interest, such that the interests of humanity at large rather than particular subgroups can potentially be considered in weighing up whether a study sufficiently advances the public interest’.^164^ Moreover, any given scenario entails an assessment of the extent of access (who is seeking access to the information, how much of it, for how long, under what conditions, etc.) and terms of access (ie, access only to those who can further the public interest), as well as a consideration of proportionality (access to confidential information is considered within the least restrictive means in the given scenario as there are no other reasonable means available to pursue the public interest-driven scientific endeavor and there is an evidenced commitment to use the information only as far as is necessary).

What the foregoing analysis demonstrates is that if, in a particular case, it is determined that there are *prima facie* grounds for confidential information disclosure on the basis of an overriding public interest, invocation of the human right to science can, as a buttress, help promote a broader view of what might be in the public interest for it to be seen as a benefit for the wider community. Yet, a series of considerations also must be factored in the analysis, supplementing the two main evaluative frameworks outlined above, namely:

1. Whether individuals have a reasonable expectation of confidentiality in respect of the relevant information, which in turn may depend, among other things, on the level of risk associated with access to and use of the relevant information for a research project (with minimal risk in the proposed research being associated with a stronger argument for a public interest basis), the perceived sensitivity of the information, and whether vulnerable populations are involved.^165^
2. Whether it is reasonably practicable to obtain consent and if not, persuasive, evidence-based reasons as to why it is not reasonably practicable.^166^ Here again, it may be argued that consent should be sought for research involving confidential information that entails greater than minimal risk.
3. Whether the research has a commercial purpose and/or the research is commercially funded (in which case the bar for disclosure likely will be even higher, but should be counterbalanced by the argument that for some groups, the only way they will reap any benefits is through commercially funded research).
4. Whether the proposed research is likely to likely to have significant public health and/or biomedical value.
5. Whether there has been delineation of the scope of restricted information disclosure as a control device: only those persons who have a proper interest in receiving confidential information should receive it and those persons, in turn, should be made aware of their obligations to hold the information in confidence to prevent unauthorized disclosures and misuse. Moreover, disclosure should be appropriately limited in its scope, duration, and extent—massive, ongoing confidential ‘data dumps’ are unlikely to meet with approval.^167^
6. Whether it is possible to either pseudonymize or anonymize any or all of the information to prevent risk of misuse and work around or limit confidential (read: identifiable) information disclosure; make use of already-existing publicly available (ie, non-confidential) information; or otherwise access and make use of the information in a security-preserving, confidentiality-enhancing manner such as a trusted research environment/secure data environment or data safe haven.^168^
7. Whether a court should authorize such disclosure by means of its general declaratory jurisdiction and to avoid future litigation risk from various parties, or whether it is sufficient for an independent oversight body or expert committee (eg, a confidentiality advisory committee) to exercise this decision, or whether it should be at the discretion of individual data custodians.
8. Whether the proposed research has been approved by a competent, independent research ethics committee; the committee has been informed that confidential information will be disclosed without consent; and the legal basis for such disclosure is the public interest.

### V.C. Analogy to Data Protection Law

As a coda, we may compare the difficult hurdles to widely expand the public interest basis in confidentiality law with data protection law, as reflected in the UK GDPR^169^ and Data Protection Act 2018. Under data protection law, personal data may be processed when it ‘is necessary for the performance of a task carried out in the public interest’^170^ and special category personal data (such as health data and genetic data), while otherwise prohibited, may be processed if, among other exemptions and conditions, there is a public interest basis in one of three areas: archiving,^171^ public health,^172^ and unspecified ‘reasons of *substantial* public interest’.^173^ Concerning this last basis, as Georgieva and Kuner note (in reference to the EU GDPR, which carries almost precisely the same language in these provisions as the UK GDPR):

Finding a substantial public interest requires a balancing between the public interest and the risks for data subjects. To process sensitive data the public interest must be ‘substantial’, in contrast to the conditions for processing personal data based on a task carried out in the public interest under [GDPR] Article 6(1)(e), where there is no requirement that the public interest be substantial. Recital 46 [of the GDPR] mentions as examples of data processing serving ‘important grounds’ of public interest ‘when processing is necessary for humanitarian purposes, including for monitoring epidemics and their spread or in situations of humanitarian emergencies, in particular in situations of natural and man-made disasters’. Voter data may be processed based on the public interest when this is required by the operation of the democratic system in a Member State, as long as appropriate safeguards are established. The threshold for satisfying this criterion is thus high.^174^

Under data protection law, too, then, we see that public health emergencies and humanitarian emergencies would likely fulfil the criteria of a (substantial) public interest basis to process sensitive (special category) personal data without seeking and obtaining consent, though even here it would require processing to be permitted on the basis of domestic law that must be proportionate to the aim pursued, respect the essence of the right to data protection, and provide for suitable and specific measures to safeguard the fundamental rights and the interests of the data subject.

If we consider the COVID-19 context, the UK’s Coronavirus Act 2020 amended primary public health legislation to enable secondary legislation to be drafted that would require persons to provide information or answer questions (including information or questions relating to their health).^175^ This would suggest that the exemption the government would rely upon to process special category personal data, such as health information, would be either the public health *or* substantial public interest basis under UK GDPR Article 9(2)(i) and 9(2)(g), respectively. Yet, it is difficult to envision how a particular human right provision, such as the human right to science, could itself form the basis of a domestic law, even as the UK is bound to the ICESCR under international law. And again, given that the UK has not agreed to be bound by the Optional Protocol to the ICESCR, which allows individuals complain to the Committee on Economic, Social and Cultural Rights about violations of their ICESCR rights, and given that the ICESCR is not directly part of UK law, in contrast with the ECHR under the HRA 1998, there is no explicit domestic law upon which a data controller—be it the government or otherwise—could rely to justify processing special category personal data on the basis of the public interest, which in turn is grounded in the human right to science. Reliance on high-level customary law and principles of common law would have to suffice, and there is legal uncertainty and risk in doing so.

## VI. CONCLUSION: THE HUMAN RIGHT TO SCIENCE AS A WEDGE TO EXPAND THE PUBLIC INTEREST BASIS?

This article has demonstrated the ostensible benefits—but also clear limitations—of relying on the public interest basis, supported by the human right to science, to lawfully permit the disclosure and use of confidential information for a research purpose. While the public interest is a malleable concept, and courts have encouraged flexibility as to its categories, it is not so elastic as to encompass a general override of confidentiality on the basis of promoting scientific advancement and its applications. Nor, arguably, would we want it to stretch *too* far, lest the relationship between patients and doctors and participants and researchers (as well as educators and pupils, and employers and employees) be scarred and informational autonomy interests irrevocably damaged—undermining the public interest in health care and health research, among other important sectors. There may be a social contract in which all of us as citizens have a responsibility to contribute to research in exchange for reaping the benefits of modern medicine,^176^ but that responsibility must be protected and respected with a number of safeguards and, ideally and where possible, undertaken with our consent. Sharing our confidential information to certain others without our consent, even in the aim of promoting scientific advancement and its applications, constitutes a significant infringement of informational autonomy and cannot stand on its own as a general category of a public interest basis.

However, what we may find is some limited judicial and juridical willingness, on a case-by-case basis, to broaden the public interest somewhat. Specifically, the argument made in this article is that there is scope to permit disclosure of confidential information to certain others and under certain conditions when there is a perceived overriding public interest basis for doing so—a public health emergency such as COVID-19, a risk of serious harm to others, and so on. More likely than not, in many scenarios, existing statutory avenues will be seen as the appropriate path forward to balance the value of data sharing and protection of confidentiality interests. In England and in the context of ‘medical research’ and use of ‘confidential patient information’, this would be ongoing resort to Regulation 5 of the COPI Regulations and reliance on CAG as an independent, expert body to assess, on an individual application basis, whether confidential patient information can be disclosed in the absence of consent. For other forms of research and categories of confidential information, the basis is more uncertain and likely subject to an even higher threshold. The legal and ethical evaluative frameworks discussed in Section 5 above offer some potential avenue for assessing when a public interest disclosure may be lawful in these contexts, particularly if combined; yet invariably, difficult judgments will need to be made and no simple solutions can be proffered.

I argue that were there a decision made by a competent authority to share confidential information on the grounds of public interest, the human right to science may be invoked as a gloss that emphasizes the importance of the informational requirement in the particular circumstances. This said, and in addition to the above qualifications, there are strong policy reasons to apply this basis narrowly. Foremost, the absence of consent means that there is greater risk of infringement of the informational autonomy interests of individuals as well as actions that fail to accord with their reasonable expectations of confidentiality. This in turn could harm the scientific endeavor if individuals, and the public *en masse*, fear that their confidential information may be misused and disclosed without their knowledge, much less acceptance. As Ballantyne and Schaefer put it, ‘The sustainability of biomedical research using identifiable health data relies on earning public trust. The research community must demonstrate that data are being managed fairly and responsibly. Public trust is enhanced by clarity, transparency and consistency.’^177^ Extra care and attention should be placed when assessing public interest disclosures for non-therapeutic research-related purposes that involve confidential information, including when the human right to science is invoked.

Even where confidential information is shared on the basis of an overriding public interest, there is an obligation to make the disclosure knowable, fair, and transparent and, where at all possible, provision made for individuals to object or refuse, and, also if possible, opt out from the disclosure. This, in turn, means there must be sufficient processes of public engagement and participation—which may be difficult in the midst of a given public health emergency, but can nonetheless be charted going forward to help map what kinds of public health emergencies (or other contexts) might be seen as acceptable. After all, the dual nature of the human right science lends weight to the importance of participation in the deliberation of such a disclosure decision and in any putative scientific endeavor. People, as citizens in a polity that relies on abstract but powerful legal constructs such as the ‘public interest’, ought to have a say in deciding whether their confidential information ought to be shared as a means to both promote the beneficial effects of science and protect against any adverse effects.

This article cannot endeavor to chart all the circumstances in which the human right to science may aid a public interest basis for disclosing confidential information. What I have done, though, is craft the contours of a conceptual argument in which we might envision an incremental expansion of the scope of the public interest in the research context, as well as an analytical framework upon which a more robust construct of the public interest basis in confidentiality law might be developed. How much an incremental expansion of the scope, if at all, may be seen as according with the reasonable expectations of the public ultimately is not only a legal question, but an ethical and socio-political one, too, that necessitates empirical investigation with robust patient and public involvement. To this end, and as an early step, research institutions should set their own internal policies clarifying for researchers how the determination of the public interest is to be undertaken and how in practice it should be evaluated.^178^ This must, of course, be drafted in alignment with the existing jurisprudence and regulatory guidance to help drive harmonization and establish precedent. But even more fundamentally, and in tune with ethics and social science input, a next research step in this area ought to be further engagement of the public to determine the scope of the public interest and reasonable expectations, the kinds of research that would reasonably be considered to contribute to the public interest, and the contours of the human right to science as a juridical buttress to enable lawful sharing of confidential information.

## DISCLOSURES

I am a member of the National Data Guardian’s Panel, which advises on the state of information governance across the health and care system in England. The views expressed in this article are my own and do not represent the views of that Panel or the National Data Guardian.

## Footnotes

1 See generally Carolyn Adams, Judy Allen, and Felicity Flack, Sharing Linked Data for Health Research: Toward Better Decision Making (2022).

2 Campbell v. Mirror Group Newspapers [2004] UKHL 22. The law of privacy in the informational context holds that where individuals have a reasonable expectation of privacy in their information, it must not be misused by others—with the caveat of unless there is a lawful justification that negates the misuse.

3 C v. Chief Constable of the Police Service of Scotland [2019] CSOH 48.

4
Lee Bygrave, Data Privacy Law: An International Perspective (2014).

5 In this article, I use the terms duty of confidentiality and duty of confidence interchangeably.

6 Coco v. AN Clark (Engineers) Ltd [1968] FSR 415.

7 Paula Case, *Confidence Matters: The Rise and Fall of Informational Autonomy in Medical Law*, 11 Med. Law Rev. 208, 213 (2003).

8 Secondary use may be defined as the use of information which is separate and distinct from, or otherwise subordinate to, the primary use of the collection or receipt. For example, patient information may be collected for the purpose of direct care of the patient; a secondary use of that information may be administration, planning, audit, or research.

9 Aileen McHarg, *Reconciling Human Rights and the Public Interest: Conceptual Problems and Doctrinal Uncertainty in the Jurisprudence of the European Court of Human Rights*, 62 Mod. L. Rev. 671 (1999); Angela Ballantyne and G. Owen Schaefer, *Public Interest in Health Data Research: Laying Out the Conceptual Groundwork*, 46 J. Med. Ethics 610 (2020). See also G. Owen Schaefer, et al., *Clarifying How to Deploy the Public Interest Criterion in Consent Waivers for Health Data and Tissue Research*, 21 BMC Med. Ethics 23 (2020).

10 Mark Taylor and Tess Whitton, *Public Interest, Health Research and Data Protection Law: Establishing a Legitimate Trade-Off between Individual Control and Research Access to Health Data*, 9 Laws 6 (2020). See also the Health and Social Care (Control of Data Processing) Act (Northern Ireland) 2016, which mandates Northern Ireland’s Department of Health to make regulations that enable the use of health and social care information which identifies individuals to be used for ‘health care or social care purposes’ which are in the public interest, without the consent of the individuals whose information may be used. The provisions of the Act will only be utilized where (i) it is impossible or impracticable to gain the consent of individuals; (ii) anonymized or pseudonymized information would not achieve the desired outcome; and (iii) the committee established under the statutory provisions authorizes the processing. As of the time of writing, due to ongoing political instability in Northern Ireland, no regulations have been promulgated, and thus, it is unclear what framework might guide assessments of secondary use health care or social care purposes that are in the public interest. I further discuss this interesting piece of legislation, which is supportive of my principal argument that the public interest can be a suitable legal basis to support a cautious widening of secondary uses of health information (and other kinds of information), later in the article.

11 See Mersey Care NHS Trust v. Ackroyd (No.2) [2006] EWHC 107 (QB), which held that institutions, such as hospitals, also have rights and responsibilities in respect of confidential information. The decision was upheld by the Court of Appeal at [2007] EWCA Civ 101.

12 See section 251 of the NHS Act 2006, which allows for the disclosure of so-called ‘confidential patient information’ under defined conditions, as set out in detail in the Health Service (Control of Patient Information) Regulations 2002 (SI 2002/1438). While section 251 applications will be covered in Section 3 of this article, it is important to note early on that I am drawing a distinction between consideration of public interest as part of an application for section 251 support (the legal basis itself being the statutory gateway)—which applies to ‘confidential patient information’—and (overriding) public interest relied outside of a section 251 application, which would apply to non-‘confidential patient information’ and where the legal basis itself is the public interest.

13 Health and Social Care (Control of Data Processing) Act (Northern Ireland) 2016, *supra* note 10. One notes that the legislation, in section 1(1), speaks only of prescribed confidential information disclosures for ‘health care or social care purposes’ and which are ‘necessary or expedient in the public interest.’ It is unclear whether this would encompass some forms of health research.

14 Éditions Plon v. France (58148/00) [2004] ECHR 200; Lewis v. Secretary of State for Health [2008] EWHC 2196.

15
Adams, et al., *supra* note 1, at 33–38, 125–127.

16
*Id*. at 142.

17
*Id*.

18 See eg, Sebastian Porsdam Mann, Julian Savulescu, and Barbara Sahakian, *Facilitating the Ethical Use of Health Data for the Benefit of Society: Electronic Health Records, Consent and the Duty of Easy Rescue*, 374 Philos. Trans. A Math. Phys. Eng. Sci. 20160130 (2016).

19 English courts still do not recognize privacy as a stand-alone common law right. See eg, Wainwright v. Home Office [2003] UKHL 53; [2004] 2 AC 406. However, as discussed later in this article, the tort of misuse of private information has been endorsed by the UK Supreme Court, suggesting a jurisprudential opening to recognizing at least some forms of a justiciable privacy right. See Bloomberg LP v. ZXC [2022] UKSC 5. In Scotland, recently there appears to be greater recognition of a fully-fledged common law right of privacy. See C v. Chief Constable of the Police Service of Scotland, *supra* note 3.

20 See *infra* note 85.

21 Neil Richards and Daniel Solove, *Privacy’s Other Path: Recovering the Law of Confidentiality*, 96 Geo. L.J. 123, 157 (2007) (‘Although the tort of breach of confidence has seen some development in recent decades, it remains in a relatively obscure and frequently overlooked corner of American tort law.’).

22
Megan Richardson, et al., Breach of Confidence: Social Origins and Modern Developments (2012).

23 47 ER 1313, (1825) 1 H & Tw 28.

24 (1849) 1 Mac & G 25, 41 ER 1171.

25 There is some authority that the action for breach of confidence is *sui generis* in nature and that it is difficult to confine the action exclusively within one conventional jurisdictional category such as contract or equity. See Tanya Aplin, et al., Gurry on Breach of Confidence: The Protection of Confidential Information (2nd edn, 2012), at paras. 4.01–4.09.

26 Attorney-General v. Guardian Newspapers (No 2) [1990] 1 AC 109 at 269, 282.

27 [1968] FSR 415.

28
*Id*. at 419.

29 (1851) 4 D 177.

30
*Id*. at 180.

31 Richards and Solove, *supra* note 21, at 160.

32 Stephens v. Avery [1988] Ch. 449; [1988] FSR 510, 517.

33 In North American parlance, these are often referred to as consent forms.

34 See eg, Public Health Scotland, *Our Privacy Notice*, https://www.publichealthscotland.scot/our-privacy-notice/personal-data-collection/ (accessed Apr. 28, 2023) (‘Depending on the type of project, the request for access [to personal data] may be scrutinised by an external independent public benefit and privacy panel which includes patient representatives. This panel checks that we protect personal data and meet our legal obligations of data protection and confidentiality.’).

35 R v. Department of Health Ex p. Source Informatics Ltd (No.1) [2000] 1 All ER 786.

36 See eg, McInerney v. MacDonald [1992] 2 SCR 138 at 148, in which La Forest J of the Supreme Court of Canada noted that ‘When a patient approaches a physician for healthcare, he or she discloses sensitive information concerning personal aspects of his or her life. […] The patient “entrusts” this personal information to the physician for medical purposes.’

37 Attorney-General v. Guardian Newspapers (No 2) [1990] 1 AC 109, 281–282 per Lord Goff: ‘detriment or potential detriment to the plaintiff will nearly always form part of his case; but this may not always be necessary’.

38 Tchenguiz v. Imerman [2010] EWCA Civ 908, esp. paras 72–79.

39 Or otherwise information that may have the necessary quality of confidence.

40 See generally CM Phipps, et al., Toulson and Phipps on Confidentiality (4th edn, 2020).

41 ABC v. St George’s Healthcare NHS Trust [2020] EWHC 455 (QB), Yip J, para 37.

42 Z v. Finland (1998) 25 EHRR 371, para 95.

43
Phipps, et al., *supra* note 40, at para 5–057.

44 McHarg, *supra* note 9, at 674 (‘There are almost as many theories of the public interest as there are writers on the subject.’). McHarg notes that the philosopher Virginia Held usefully identified three general categories into which different versions of the public interest may fall: preponderance or aggregative theories, unitary theories, and common interest theories. See Virginia Held, The Public Interest and Individual Interests (1970). McHarg rejects preponderance theories of the public interest because, among other reasons, ‘it involves sacrificing the interests of the few to those of the many, regardless of the merits of their respective claims measured against other standards’ (p. 675), and unitary theories, too, because they ‘place *too much* faith in the possibility of transcending apparent conflicts between individual interests’ and can slide into paternalism (p. 675). She lends more support to common interest theories, that is, ‘interests which all members of the public have in common, hence comprising a category of interests *distinct from* those of particular individuals or groups’ (p. 676), although she also finds that common interest theories can accommodate a weaker conception of human rights than unitary theories as they are ‘impossible to reconcile with a meaningful conception of a right—as a trump over collective interests’ (p. 683). I pick up on common interest theories of the public interest later in the article.

45 See eg, Frank Sorauf, *The Public Interest Reconsidered*, 19 J. Pol. 619 (1957); Mike Feintuck, The Public Interest in Regulation (2004). Feintuck’s focus on ‘the use of the concept of the public interest as a justification for regulatory intervention into private activity, limiting the exercise of private power, in pursuit of objectives valued by community’ (p. 6), has much resonance for the analysis in this article.

46
Feintuck, *supra* note 45, at 33.

47 McHarg, *supra note* 9, at 676, paraphrasing the definition of public interest from Brian Barry, Political Argument (1990).

48 Taylor and Whitton, *supra* note 10, at 12.

49 (1856) 26 LJ Ch113 at 114 per Page-Wood VC.

50
Scottish Law Commission, Breach of Confidence: Report on a Reference Under Section 3(1)(e) of the Law Commissions Act 1965 (Scot. Law Com. No. 90, 1984), at para 4.64.

51 See eg, Beloff v. Pressdram Ltd [1973] 1 All ER 241; Lion Laboratories Ltd v. Evans [1985] QB 526; ABC v. Telegraph Media Group Ltd [2018] EWCA Civ 2329.

52
Scottish Law Commission, *supra* note 50, at para 4.67.

53 [1990] 1 AC 109 at 282–283 (emphasis added).

54
Phipps, et al., *supra* note 40, at para 5–082.

55 [2001] QB 424.

56
Phipps, et al., *supra* note 40, at para 5–083.

57 [1989] Ch 359.

58
*Id*. at 422.

59
*Id*. at 416 (emphasis added).

60 [1988] RPC 379.

61 General Medical Council, *Confidentiality: Good Practice in Handling Patient Information* (2017), https://www.gmc-uk.org/ethical-guidance/ethical-guidance-for-doctors/confidentiality (accessed Feb. 2, 2023). This version is substantially equivalent to the version cited in *Egdell*.

62 [2020] EWHC 455 (QB).

63
Phipps, et al., *supra* note 40, at para 5–113.

64 Paul Snelling and Oliver Quick, *Confidentiality and Public Interest Disclosure: A Framework to Evaluate UK Healthcare Professional Regulatory Guidance*, 22 Med. Law Int. 3 (2022).

65 Department of Health, *Confidentiality: NHS Code of Practice* (2003), https://www.gov.uk/government/publications/confidentiality-nhs-code-of-practice (accessed Feb. 2, 2023).

66 Department of Health, *Confidentiality: NHS Code of Practice—Supplementary Guidance: Public Interest Disclosures* (2010), https://www.gov.uk/government/publications/confidentiality-nhs-code-of-practice/

67 General Medical Council, *Confidentiality*, *supra* note 61.

68 GMC, General Dental Council, Nursing and Midwifery Council, General Chiropractic Council, General Optical Council, General Osteopathic Council, General Pharmaceutical Council, Health and Care Professions Council, and Social Work England.

69 Snelling and Quick, *supra* note 64, at 26.

70 Department of Health, *supra* note 65, at 6 (emphasis added).

71 See eg, Mark Taylor, *Confidentiality and Data Protection*, in Principles of Medical Law 643–711 (Judith Laing and Jean McHale eds., 2017).

72 [1978] AC 171, 230.

73 [2001] QB 424, 444.

74 [2008] EWHC 2196.

75
*Id*. at para 58.

76
*Id*. at para 59.

77 Hereinafter NHS Act 2006.

78 Lewis (*supra* note 74), at para 52.

79
*Id*. at para 54.

80
*Id*. at para 55.

81 [2001] QB 424.

82 Specifically, the general practitioner’s name, the date of prescription, the product prescribed, and the quantity prescribed. The patient’s identity was explicitly excluded from the information release.

83 [2001] QB 424, 444.

84
Phipps, et al., *supra* note 40, at para 5–057.

85 Here (and as alluded to in the introduction to this article), it is worth noting that Scotland has not proceeded to take a similarly restrictive view of the common law. There is no section 251 support equivalent in Scotland. It continues to permit disclosures of confidential information on a common law basis, albeit, in practice and in the context of confidential patient information, through an expert committee governance approach. See the work of the Public Benefit and Privacy Panel for Health and Social Care (PBPP): https://www.informationgovernance.scot.nhs.uk/pbpphsc/ (accessed Apr. 28, 2023). The PBPP is a governance structure of NHS Scotland that scrutinizes and considers applications for access to NHS Scotland health data for non-direct care purposes, such as research. The PBPP balances public benefit with potential risk to privacy and ensures that the public interest will be furthered by the proposal, detailed in an application, and demonstrates that the social need for the processing of the data requested will result in a reasonable likelihood that it will result in a tangible benefit for society. For contexts beyond confidential patient information or requested access to NHS Scotland health data, Scotland and England are by and large similar: a non-statutory gateway would be required—be it consent, the public interest, or another lawful basis. As noted in *supra* note 10, Northern Ireland appears to have adopted a broader, flexible approach, not limited to the context of confidential patient information, although also adopting an expert committee governance approach as in Scotland and limiting the context to ‘health care or social care purposes’. The Northern Ireland Assembly passed the Health and Social Care (Control of Data Processing) Act (Northern Ireland) 2016 (albeit without the necessary regulations to date) that permits the setting aside of the common law duty of confidentiality, where gaining individuals consent is impossible or impracticable and the use of anonymized or pseudonymized information would not achieve the desired outcome, for secondary uses of confidential health and social care information that are in the public interest. The legislation places an obligation on Northern Ireland’s Department of Health to make regulations to establish a committee authorize processing of confidential information and the dissemination of information. It remains to be seen if the necessary secondary legislation will be established in the coming years to bring to fruition this system of public interest-based confidential information disclosure for health care or social care purposes.

86 Case, *supra* note 7, at 219, 221 (‘The problem with such an application [of the public interest basis to medical research] is that authorities on the public interest defence deal with specific, identifiable and immediate risks to the public rather than potential or generalised risks, such as those posed by not pursuing potential medical advances by research. […] The common law’s position on this issue is far from clear, but neither implied consent nor the public interest defence appear to be sufficiently robust to afford protection to medics who disclose patient information for the purposes of medical research or general public health surveillance’).

87 The HRA is an arm’s length body of the Department of Health and Social Care and serves as a regulator of different aspects of health and social care research. Most of its functions apply to research undertaken in England, but it also works closely with the other nations in the UK to provide a UK-wide harmonised system of health research regulation.

88 Health Research Authority, *What is Confidential Patient Information and Why is it Used?*, https://www.hra.nhs.uk/about-us/committees-and-services/confidentiality-advisory-group/confidential-patient-information-and-regulations/ (accessed Apr. 28, 2023). A previous version of this web page in 2022 phrased the explanation more explicitly: ‘Section 251 […] came about as it was recognised that there were essential activities of the NHS and medical research that required the use of identifiable patient information. However, because patient consent had not been obtained to use patients’ confidential information for these other purposes, *there was no secure basis in law for doing so*.’ (emphasis added).

89 Annie Sorbie, *Sharing Confidential Health Data for Research Purposes in the UK: Where are ‘Publics’ in the Public Interest?*, 16 Evid. & Pol’y 249 (2019).

90 Michel Coleman, Barry Evans, and Geraldine Barrett, *Confidentiality and the Public Interest in Medical Research*—*Will We Ever Get It Right?*, 3 Clin. Med. 219 (2003); Joan Higgins, *The Patient Information Advisory Group and the Use of Patient-Identifiable Data*, 8 Suppl. 1 J. Health Serv. Res. Pol’y (2003).

91 Section 251(10), NHS Act 2006 (emphasis added).

92 Section 251(11), NHS Act 2006.

93 Health Service (Control of Patient Information) Regulations 2002 (SI 2002/1438), reg 4.

94 Health Research Authority, *Confidentiality Advisory Group*, https://www.hra.nhs.uk/about-us/committees-and-services/confidentiality-advisory-group/ (accessed Apr. 28, 2023).

95 Section 6 of the Health and Social Care Act 2012, inserting section 1E into the National Health Service Act 2006.

96 The HSCIC subsequently became brand named as NHS Digital. In Feb. 2023, it was subsumed within NHS England under the Health and Social Care Information Centre (Transfer of Functions, Abolition and Transitional Provisions) Regulations 2023.

97 This information was available on a previous version of the Health Research Authority webpage, *FAQs About the Law*, https://s3.eu-west-2.amazonaws.com/www.hra.nhs.uk/media/documents/cag-frequently-asked-questions-1.pdf (accessed Apr. 28, 2023). See more recently, Integrated Research Application System, *Confidentiality Advisory Group: Validation Criteria*, https://www.myresearchproject.org.uk/help/hlpconfidentiality.aspx#Validation-Criteria (accessed Apr. 28, 2023).

98 Department of Health and Social Care, [*withdrawn] Coronavirus (COVID-19): Notice Under Regulation 3(4) of the Health Service (Control of Patient Information) Regulations 2002*—*general* (2022), https://www.gov.uk/government/publications/coronavirus-covid-19-notification-of-data-controllers-to-share-information/coronavirus-covid-19-notice-under-regulation-34-of-the-health-service-control-of-patient-information-regulations-2002-general--2 (accessed Apr. 28, 2023).

99
*Id*.

100 Department of Health and Social Care, *Notice under Regulation 3(4) of the Health Service (Control of Patient Information) Regulations 2002* (2022), https://www.gov.uk/government/publications/covid-19-notification-to-gps-and-nhs-england-to-share-information/notice-under-regulation-34-of-the-health-service-control-of-patient-information-regulations-2002 (accessed Apr. 28, 2023).

101 As evidence of this, see NHS England, *Health Service (Control of Patient Information) Regulations 2002 (COPI) COPI Notice Exit Guidance: Guidance for Organisations on Processing of Confidential Patient Information When the COPI Notices Expire* (v. 2, June 8, 2022) *supra* note 6, https://digital.nhs.uk/services/data-access-request-service-dars/copi-guidance (accessed Apr. 28, 2023) (‘We understand that Regulation 3 may be used as a legal basis for processing [confidential patient information] for diagnosing communicable diseases, recognising trends, controlling, and preventing the spread and monitoring and managing communicable disease (Regulation 3(1)). However, as we move out of the pandemic period, our view is that increasingly *fewer research projects will meet the requirements of Regulation 3(1)* and, as such, Regulation 5 is more likely to be the appropriate legal basis for ongoing and future COVID-19 research.’) (emphasis added).

102 Department of Health, *supra* note 66, at para 2.

103
*Id*. at para 3.

104
*Id*. at para 17.

105
*Id*. at *supra* note 12.

106 Ballantyne and Schaefer note, for example: ‘Empirical research shows that [research ethics committees] find interpreting public interest on behalf of a pluralistic community stressful and ambiguity regarding the scope and meaning of these terms contributes to this stress.’ See Ballantyne and Schaefer, *supra* note 9, at 611.

107 British Medical Association, *Confidentiality Toolkit: A Toolkit for Doctors* (2021), https://www.bma.org.uk/advice-and-support/ethics/confidentiality-and-health-records/confidentiality-and-health-records-toolkit (accessed Apr. 28, 2023).

108
*Id*. at 14.

109
*Id*.

110
*Id*. at 16.

111
*Id*. at 14 (emphasis added).

112 Caldicott Guardians are senior individuals in health and social care organizations in the UK responsible for ensuring that confidential information about health and social care service users is used ethically, legally, and appropriately. See the UK Caldicott Guardian Council (UKCGC), https://www.ukcgc.uk/ (accessed Apr. 28, 2023). See also National Data Guardian, *Guidance about the Appointment of Caldicott Guardians, Their Role and Responsibilities Published by the National Data Guardian for Health and Social Care* (2021), https://www.gov.uk/government/publications/national-data-guardian-guidance-on-the-appointment-of-caldicott-guardians-their-role-and-responsibilities (accessed Apr. 28, 2023).

113 NHS Digital, *NHS Digital Statement on Health Select Committee’s Report into Patient Data Sharing (Apr. 18, 2018)*, https://digital.nhs.uk/news/2018/nhs-digital-statement-on-health-select-committees-report-into-patient-data-sharing (accessed Apr. 28, 2023).

114 See *Letter from the Chair of the Health and Social Care Select Committee to the Chief Executive of NHS Digital, Jan. 29, 2018*, and House of Commons Health and Social Care Committee, *Memorandum of Understanding on Data-Sharing Between NHS Digital and the Home Office, Fifth Report of Session 2017–19*, https://publications.parliament.uk/pa/cm201719/cmselect/cmhealth/677/67702.htm (accessed Apr. 28, 2023). The letter and report from the Health and Social Care Select Committee demonstrate a concern regarding a memorandum of understanding between NHS Digital, the Home Office, and the Department of Health (which came into effect in Jan. 2017) on processing information requests from the Home Office to NHS Digital for tracing immigration offenders. The memorandum allowed the Home Office to gain access to patient data, including non-clinical demographic information, for the purpose of tracing immigration offenders and vulnerable people. NHS Digital, in turn, could be asked to hand over information such as a patient’s date of birth or last known address to the Home Office. It is understood that around 3000 NHS patients’ details were shared with the Home Office in 2017 so they could check those people’s immigration status. The Committee argued that the public interest in the disclosure of information held by the NHS was heavily outweighed by the public interest in the maintenance of a confidential medical service. The data sharing arrangement and underlying memorandum of understanding were abandoned shortly after the Health and Social Care Select Committee’s report. Henceforth, the Home Office immigration staff would only be able to use the data sharing mechanism to trace people being considered for deportation from the United Kingdom because they have committed a serious crime. See also Hannah Crouch, *NHS Digital’s Sharing of Non-Clinical Patient Data Branded ‘Inappropriate’* (Apr. 17, 2018), digitalhealth, https://www.digitalhealth.net/2018/04/nhs-digitals-information-sharing-home-office-inappropriate/ (accessed Apr. 28, 2023); Denis Campbell, *NHS Will No Longer Have To Share Immigrants’ Data With Home Office* (9 May 2018) The Guardian, https://www.theguardian.com/society/2018/may/09/government-to-stop-forcing-nhs-to-share-patients-data-with-home-office (accessed Apr. 28, 2023).

115 Department of Health and Social Care, *The NHS Constitution for England* (updated 1 January 2021), available at: https://www.gov.uk/government/publications/the-nhs-constitution-for-england/the-nhs-constitution-for-england (accessed Apr. 28, 2023).

116 Department of Health, *supra* note 66, at para 17.

117 Case, *supra* note 7, at 219.

118 The tort of misuse of private information was first recognized in Campbell v. Mirror Group Newspapers [2004] UKHL 22 and was affirmed as a stand-alone cause of action by the Supreme Court in Bloomberg LP v. ZXC [2022] UKSC 5. In the Court of Appeal case of Tchenguiz v. Imerman [2010] EWCA Civ 908, Lord Neuberger MR stated at paras 65–66 that, ‘there is now a tort of misuse of private information’ and that ‘there are dangers in conflating the developing law of privacy under art 8 and the traditional law of confidence’, but stated as well that ‘a reasonable expectation’—whether of privacy or confidentiality—is an appropriate test to apply when considering either cause of action. In *infra* note 159, I suggest there is some merit in this claim—not for the purpose of fulfilling the legal test for breach of confidence (which I consider to still be the modified tripartite *Coco* test)—but for the purpose of considering the legal and ethical merit of invoking the public interest, buttressed by the human right to science, as a basis to disclose confidential information.

119 Article 8(2) ECHR states: ‘There shall be no interference by a public authority with the exercise of this right except such as is in accordance with the law and is necessary in a democratic society in the interests of national security, public safety or the economic well-being of the country, for the prevention of disorder or crime, for the protection of health or morals, or for the protection of the rights and freedoms of others.’

120 [2022] UKSC 5.

121 In re S (FC) (a child) [2004] UKHL 47, para 17 (emphasis in original).

122 [2006] EWCA Civ 1776. It must be stressed that this case involved a breach of confidence claim rather than a claim for misuse of private information and arose at a time when the pioneering Campbell case (*supra* note 2) from the House of Lords was decided only two years prior. Lord Phillips observed (at para 28) that: ‘There is an issue in this case as to whether the information disclosed was private so as to engage Article 8 and there is an obvious overlap between this question and the question of whether the information was capable of being the subject of a duty of confidence under the old law. Assuming that it was, there are in this action all the elements of a claim for breach of confidence under that law. The information was disclosed in breach of a well recognised relationship of confidence, that which exists between master and servant. Furthermore, the disclosure was in breach of an express contractual duty of confidentiality.’ Arguably, this kind of analysis, distinguishing between breach of confidence claims under ‘the old law’ and ones under ‘the new law’ leaves something to be desired and would feature less (if at all) in breach of confidence cases today, where the traditional, well-established tripartite test from *Coco* (*supra* note 27) would in my view continue to apply, and the Article 8 ECHR analysis would be applied to a misuse of private information claim. The recent UK Supreme Court case of Bloomberg *LP v ZXC* [2022] UKSC 5 confirms that the tort of misuse of private information ‘is a distinct cause of action from breach of confidence’ and ‘rests on different legal foundations and protects different interests’ (Lord Hamblen and Lord Stephens, para 45) but, unfortunately, the Court did not confirm whether the modified tripartite test from *Coco* is still in fact the correct legal test to apply.

123
*Id*. at paras 67–68.

124 [1990] 1 AC 109 at 283–284.

125 The test is: would a conscientious recipient of confidential information reasonably consider it right as a responsible citizen to make some form of disclosure of the information?

126
Phipps, et al., *supra* note 40, at para 5–100.

127 See generally Samantha Besson, *Human Rights in Relation: A Critical Reading of the ECtHR’s Approach to Conflicts of Rights*, in When Human Rights Clash at the European Court of Human Rights: Conflict or Harmony? 23–27 (Stijn Smet and Eva Brems eds., 2017).

128 See generally Helle Porsdam and Sebastian Porsdam Mann (eds.), The Right to Science: Then and Now (2021).

129 Rumiana Yotova and Bartha Knoppers, *The Right to Benefit from Science and Its Implications for Genomic Data Sharing*, 31 Eur. J. Int’l L. 665 (2020). See also Shawn Harmon, *A Tale of Two Legacies: Drawing on Humanist Interpretations to Animate the Right to the Benefits of Science*, in Law and Legacy in Medical Jurisprudence: Essays in Honour of Graeme Laurie 391–416 (Edward Dove and Niamh Nic Shuibhne eds., 2022). This is not to say that the UK as a State fails to support scientific advancement and its benefits; it is a trite observation that the UK is one of the world’s leading science superpowers and this is partly due to long-standing government support through favorable funding and regulatory structures.

130
*Id*.

131 For the pyramidic relationship between data, information, and knowledge, see generally Rob Kitchin, The Data Revolution: A Critical Analysis of Big Data, Open Data and Data Infrastructures (2nd edn, 2022).

132 Yotova and Knoppers, *supra* note 129, at 690.

133
United Nations General Assembly—Human Rights Council, Report of the special rapporteur in the field of cultural rights: the right to enjoy the benefits of scientific progress and its applications (United Nations: Human Rights Council, Twentieth Session, A/HRC/20/26, 2012) 1.

134
*Id*. at 8.

135 Article 4 ICESCR states: ‘The States Parties to the present Covenant recognize that, in the enjoyment of those rights provided by the State in conformity with the present Covenant, the State may subject such rights only to such limitations as are determined by law only in so far as this may be compatible with the nature of these rights and solely for the purpose of promoting the general welfare in a democratic society.’

136
United Nations General Assembly—Human Rights Council, *supra* note 133, at 13–14.

137
*Id*. at 14.

138
United Nations General Assembly—Human Rights Council, Report of the United Nations High Commissioner for Human Rights: Report on the seminar on the right to enjoy the benefits of scientific progress and its applications (United Nations: Human Rights Council, Twenty-Sixth Session, 2014, A/HRC/26/19) 6.

139
United Nations Economic and Social Council—Committee on Economic, Social and Cultural Rights, General comment No. 25 (2020) on science and economic, social and cultural rights (article 15(1)(b), (2), (3) and (4) of the International Covenant on Economic, Social and Cultural Rights) (United Nations: Committee on Economic, Social and Cultural Rights, 2020, E/C.12/GC/25) 2.

140
*Id*. at 5.

141
*Id*.

142 Yotova and Knoppers, *supra* note 129, at 671.

143 Cesare Romano, *The Origins of the Right to Science: The American Declaration on the Rights and Duties of Man*, in The Right to Science: Then and Now 33–53 (Helle Porsdam and Sebastian Porsdam Mann, eds., 2022).

144
Committee on Economic, Social and Cultural Rights, General comment No. 25, *supra* note 139, at 19.

145 Yotova and Knoppers, *supra* note 129, reference only two cases on point: Supreme Court of Venezuela, López, Glenda y otros c. Instituto Venezolano de los Seguros Sociales, Sentencia no. 487 (2001) and Inter-American Court of Human Rights, Artavia Murillo v. Costa Rica, Judgment (Preliminary Objections, Merits, Reparations, and Costs), Nov. 28, 2012.

146 Ministry of Justice (UK Government), *International Covenant on Economic, Social and Cultural Rights (ICESCR): 7th Periodic Report* (May 2022), https://www.gov.uk/government/publications/international-covenant-on-economic-social-and-cultural-rights-icescr-7th-periodic-report (accessed Apr. 28, 2023).

147
*Id*. at para 2.

148
*Id*. at para 179.

149 United Kingdom, First Periodic Report, Doc. E/1990/7/Add.16, Nov. 24, 1993, quoted in Yotova and Knoppers, *supra* note 127.

150 Yotova and Knoppers, *supra* note 129, at 680.

151
*Id*.

152
*Id*. at 685.

153 See eg, Understanding Patient Data, *Understanding Public Expectations of the Use of Health and Care Data* (2019), https://understandingpatientdata.org.uk/sites/default/files/2019-07/Understanding%20public%20expectations%20of%20the%20use%20of%20health%20and%20care%20data.pdf (accessed Apr. 28, 2023). See also National Data Guardian, *Putting Good into Practice: A Public Dialogue on Making Public Benefit Assessments When Using Health and Care Data* (2021), https://www.gov.uk/government/publications/putting-good-into-practice-a-public-dialogue-on-making-public-benefit-assessments-when-using-health-and-care-data (accessed Apr. 28, 2023).

154 See NHS Digital, *Data Security and Protection Toolkit*, https://www.dsptoolkit.nhs.uk/ (accessed Apr. 28, 2023).

155 National Data Guardian, *What Do We Mean by Public Benefit? Evaluating Public Benefit When Health and Adult Social Care Data is Used for Purposes Beyond Individual Care* (2022), https://www.gov.uk/government/publications/what-do-we-mean-by-public-benefit-evaluating-public-benefit-when-health-and-adult-social-care-data-is-used-for-purposes-beyond-individual-care (accessed Apr. 28, 2023).

156
*Id*. at 3.

157
*Id*. at 8.

158
*Id*. at 11. The guidance includes a series of questions organizations should consider to determine whether their proposed data use might have associated risks that could outweigh public benefit.

159 I deliberately use the term ‘reasonable expectation of confidentiality’, rather than ‘reasonable expectation of privacy’, to avoid conflating these two concepts and the relevant legal tests that apply to each cause of action, viz. breach of confidence and misuse of private information. Nonetheless, there are important similarities that ought to be considered. As Lord Hamblen and Lord Stephens noted in Bloomberg LP v. ZXC [2022] UKSC 5 at para 150 (emphasis added): The recognition that the causes of action for misuse of private information and for breach of confidence are distinct means that there is no necessary overlap between them. Information may be private but not confidential, or confidential but not private. To prove that information is private it is not necessary to show that it is confidential. *Often, however, confidentiality and privacy will overlap and confidentiality may well be relevant to whether there is a reasonable expectation of privacy. In particular, if information is confidential that is likely to support the reasonableness of an expectation of privacy*. Although the modified tripartite legal test to establish whether there is a breach of confidence does not per se require looking at reasonable expectations (as discussed in Section II above), it is arguable that in the context of invoking the public interest, buttressed by the human right to science, as a basis to disclose and access confidential information, one ought to consider the confider’s reasonable expectations of confidentiality in the relevant information, with the expectation being that of a reasonable person of ordinary sensibilities placed in the same position as the confider. If there is credence to my argument, further research ought to consider whether, and if so how, the evaluation of reasonable expectations of confidentiality might differ from reasonable expectations of privacy, such as focusing more on the circumstances giving rise to an obligation of confidence, such that information is in effect impressed with a duty of confidence owed to the person who has entrusted it. For example, the evaluation might consider the circumstances of the information being imparted and being accepted by the confidant, asking whether it was reasonable to infer that it was on the basis that the information would be kept secret.

160 Rebecca Haffajee, Wendy Parmet, and Michelle Mello, *What Is a Public Health ‘Emergency’?*, 371 New Eng. J. Med 986 (2014). One could also accept a paraphrased definition from the World Health Organization’s concept of public health emergency of international concern, defined in the 2005 International Health Regulations, as an extraordinary event which is determined to constitute a public health risk to a given jurisdiction through the spread of disease and which potentially requires a coordinated response from the state.

161 See *supra* note 47.

162 Schaefer, et al., *supra* note 9, at 4.

163
*Id*. at 5.

164
*Id*. at 615.

165 See eg, Porsdam Mann, et al., *supra* note 18, who argue that the ‘duty of easy rescue’ rescue—the principle that persons should benefit others when this can be done at no or minimal risk to themselves—grounds the removal of consent requirements for minimally risky records-based research, particularly in the context of electronic health records.

166 For in-depth research on this issue, see Sara Laurijssen, et al., *When Is It Impractical to Ask Informed Consent? A Systematic Review*, 19 Clin. Trials 545 (2022). The authors found there to be four different conditions described as impracticability: (i) obtaining informed consent becomes too demanding for researchers, (ii) obtaining informed consent leads to invalid study outcomes, (iii) obtaining informed consent harms the participant, and (iv) obtaining informed consent is meaningless for the participant. The authors argue that researchers have a moral responsibility to design an infrastructure in which consent can be obtained, even if they face hardship in obtaining consent, and that researchers should seek to minimize harm inflicted upon participants when harm may occur as a result of the consent procedure.

167 See also Saab v. Dangate Consulting Ltd [2019] EWHC 1558 (Comm).

168 On the benefits of trusted research environments, see generally Ben Goldacre, et al., Better, Broader, Safer: Using Health Data for Research and Analysis (2022), known as the ‘Goldacre Review’, https://www.goldacrereview.org/ (accessed Apr. 28, 2023). See also NHS Digital, *Secure Data Environment Service*, https://digital.nhs.uk/services/secure-data-environment-service (accessed Apr. 28, 2023).

169 Regulation (EU) 2016/679 of the European Parliament and of the Council of Apr. 27, 2016 on the protection of natural persons with regard to the processing of personal data and on the free movement of such data (United Kingdom General Data Protection Regulation) (hereinafter, UK GDPR).

170 Art. 6(1)(e), UK GDPR.

171 Art. 9(2)(j), UK GDPR.

172 Art. 9(2)(i), UK GDPR.

173 Art. 9(2)(g), UK GDPR (emphasis added).

174 Ludmila Georgieva and Christopher Kuner, *Article 9. Processing of Special Categories of Personal Data*, in The EU General Data Protection Regulation (GDPR): A Commentary 379 (Christopher Kuner, Lee Bygrave, and Christopher Docksey eds., 2020).

175 See eg, section 25B(2)(g) of Schedule 18 and section 4(2)(g) of Schedule 19 of the Coronavirus Act 2020.

176 See eg, G. Owen Schaefer, Ezekiel Emanuel, and Alan Wertheimer, *The Obligation to Participate in Biomedical Research*, 302 JAMA 67 (2009).

177 Ballantyne and Schaefer, *supra* note 9, at 611.

178 This is a point of emphasis Schaefer, et al. also stress in their article (*supra* note 9).

